# An shRNA kinase screen identifies regulators of UHRF1 stability and activity in mouse embryonic stem cells

**DOI:** 10.1080/15592294.2022.2044126

**Published:** 2022-03-24

**Authors:** Michael D. Rushton, Emily A. Saunderson, Hemalvi Patani, Michael R. Green, Gabriella Ficz

**Affiliations:** a Centre for Haemato-Oncology, Barts Cancer Institute, Queen Mary University of London, London, UK; b Horizon Discovery, Cambridge Research Park, 8100 Beach Dr, Waterbeach, Cambridge, CB25 9TL; c Research And Development, CS Genetics Ltd, Cambridge, UK; d Department of Molecular, Cell and Cancer Biology, University of Massachusetts Chan Medical School, Worcester, Massachusetts, USA

**Keywords:** DNA methylation maintenance, UHRF1, embryonic stem cells, signalling and metabolism, DNA demethylation

## Abstract

Propagation of DNA methylation through cell division relies on the recognition of methylated cytosines by UHRF1. In reprogramming of mouse embryonic stem cells to naive pluripotency (also known as ground state), despite high levels of *Uhrf1* transcript, the protein is targeted for degradation by the proteasome, leading to DNA methylation loss. We have undertaken an shRNA screen to identify the signalling pathways that converge upon UHRF1 and control its degradation, using UHRF1-GFP fluorescence as readout. Many candidates we identified are key enzymes in regulation of glucose metabolism, nucleotide metabolism and Pi3K/AKT/mTOR pathway. Unexpectedly, while downregulation of all candidates we selected for validation rescued UHRF1 protein levels, we found that in some of the cases this was not sufficient to maintain DNA methylation. This has implications for development, ageing and diseased conditions. Our study demonstrates two separate processes that regulate UHRF1 protein abundance and activity.

## Introduction

DNA methylation is the most characterized epigenetic modification with essential roles in mammalian development, ageing, cancer and other human diseases [1,2]. Inheritance of the DNA methylation patterns during cell division is a process whereby pre-existing methylation patterns on the old DNA strand are faithfully copied through a semiconservative mechanism to the newly synthesized strand in the replicating DNA [3]. Ubiquitin-like with PHD and RING finger domains 1 (UHRF1) is a central hub for this process, whereby the SRA domain of UHRF1 recognizes hemimethylated CpGs [4–6] and the presence of H3K9me2 [5] in the replicating chromatin followed by the recruitment of DNMT1 to execute the transfer of the methyl group onto the newly added cytosine. Early mammalian development is associated with large fluctuations of DNA methylation, where both paternal and maternal genomes are erased of this modification, resulting in a demethylated genome in the embryo before implantation, but rapidly re-established around embryo implantation [7]. Recent advances in *in vitro* culturing of mouse embryonic stem (mESCs), these cells have emerged as a paradigm in understanding the mechanism of DNA methylation maintenance, because they can recapitulate the hypomethylated state of the pre-implantation embryo by inhibiting the FGF2-MEK1/2 signalling pathway in cultured embryonic stem cells [8,9]. These two studies have established a strong link between signalling pathways and epigenetic regulation, which is the basis of the current study. As detailed below, dependence on signal inhibition was a feature of mESCs from their first derivation from mouse embryos. Pluripotent stem cells were first isolated directly from the pre-implantation epiblast of the mouse embryo in culture conditions optimized for teratocarcinoma stem cells [10,11]. Under these conditions mouse embryonic stem cells (mESCs) proliferate indefinitely *in vitro* on a feeder layer of mitotically inactivated fibroblasts, retain the ability to differentiate into all three germ layers, and when re-introduced into blastocysts they contribute to every tissue including gametes [12,13]. Discovery of the leukaemia-inhibitory factor (LIF) eliminated the need to use a feeder layer which secretes the LIF cytokine [14–16]. Culturing mESCs in the presence of LIF was the first step towards creating defined culture media for mESCs, even though it needed complementation with either serum or bone morphogenetic protein (BMP) to self-renew [17,18]. However, mESCs cultured in serum/LIF are heterogeneous morphologically, epigenetically [19,20], and are characterized by a fluctuating expression of pluripotency factors such as Nanog, Rex1, Stella, Esrrb and Klf4 [21–24]. Furthermore, whilst the epigenome of the inner cell mass (ICM) is characterized by global DNA hypomethylation [25,26], mESCs in serum/LIF have a highly methylated genome with around 80% CpGs methylated, which more closely resembles the methylome of adult somatic cells [8]. Given the fact that epigenetic reprogramming including DNA hypomethylation is a key hallmark in the re-acquisition of pluripotency during development, the hypermethylated status of cultured mESCs represents somewhat of a paradox. It is now considered that mESCs cultured in serum/LIF are in a state of flux – receiving pro-differentiation signals from autocrine FGF4 signalling which is attenuated by LIF – and may capture a state of pluripotency defined as ‘formative,’ which is situated between naïve pluripotency and definitive lineage serum/LIF states like the post-implantation epiblast [27]. Inhibition of FGF signalling coupled with the inhibition of glycogen synthase kinase (Gsk3b) using 2 small molecule inhibitors (2i, FGF – PD0325921, Gsk3 – CHIR99021) can drive mESCs into ground state of pluripotency [28]. 2i cultured mESCs are believed to be a more accurate reflection of the conditions of the ICM, with homogenous expression of pluripotency factors [29]. These cells, transitioned to 2i culturing, also undergo epigenetic reprogramming and are characterized by global DNA hypomethylation as well as redistribution of various histone modifications [8,30,31], representing in vivo inner cell mass cells more accurately [25,26]. Following this it has been revealed that the global loss of DNA methylation during the transition to the ground state is a consequence of the impairment of the DNA methylation maintenance machinery – specifically the loss of the DNMT1 binding partner UHRF1 at the protein level in addition to global loss of H3K9me2 [9]. It has also been identified that Pramel7 is a factor interacting directly with UHRF1 in mouse embryonic stem cells, and thereby controlling its degradation [32]. Whilst it is known that inhibition of FGF and Gsk3 signalling drives the process of DNA demethylation, the exact shift in signalling that occurs downstream of the 2i inhibition and impacts on UHRF1 is not known. In this study we set out to characterize the pathways that act downstream of 2i and are required to mediate UHRF1 downregulation. To do this we utilized a high-throughput shRNA screen using GFP expression as readout in a mESC cell line expressing a UHRF1-GFP.

## Results

### UHRF1 loss during the transition to the naïve state of pluripotency is mediated by the proteasome

**Figure 1.**
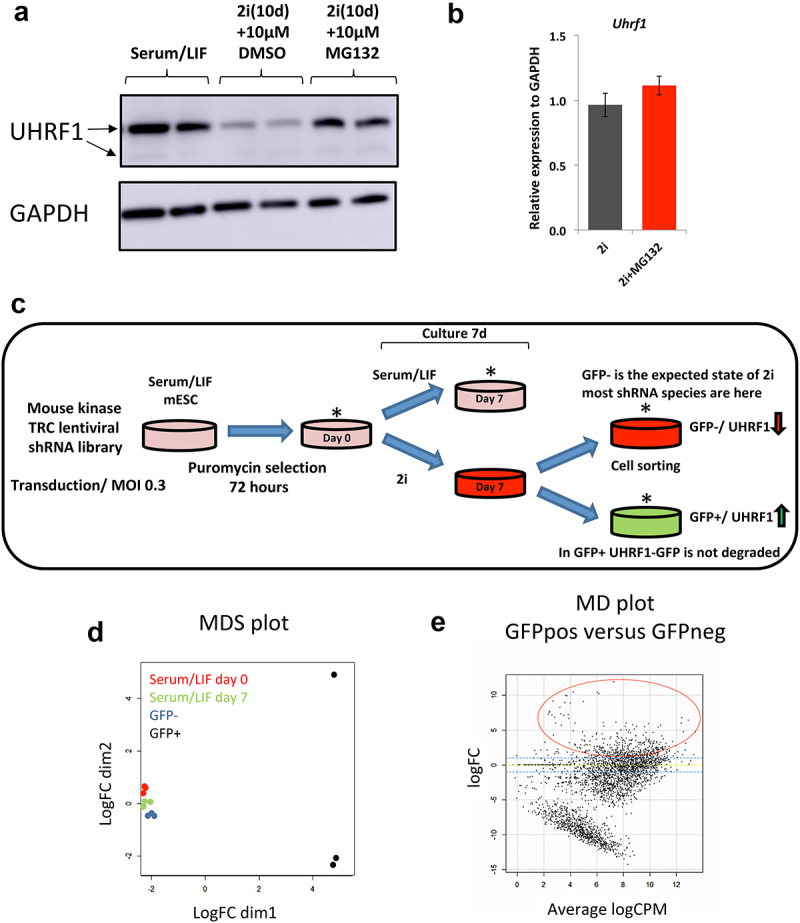
High-throughput shRNA screen-identified kinases mediating loss of UHRF1 in Serum/LIF to 2i transition in mESCs. a) Loss of UHRF1 in the transition to ground state pluripotency is mediated by the proteasome. ESCs grown in 2i conditions for a total of 10 days were subsequently cultured in the presence of the 26S proteasome inhibitor MG132 at a final concentration of 10 μM for a total of 6** **hours. Western blot analysis was performed for 2x replicates of Serum/LIF, 2i+DMSO and 2i+MG132 ESCs. Note full length UHRF1 has 2 bands indicated by arrows with the slower migrating band considered to be a modified form of UHRF1. b) MG132 does not impact UHRF1 at the transcriptional level. Data are presented as 2− ΔΔCt with Gapdh serving as a loading control and the relative expression levels of Uhrf1 in 2i and 2i+MG132 ESCs are normalized to the level of expression in Serum/LIF ESCs. Error bars represent the standard error of the mean from 3 replicates. c) Schematic detailing the experimental design for the screen, asterisks denotes samples that were collected for subsequent sequencing – Serum/LIF day 0, Serum/LIF day 7, 2i day 7 GFP+ and 2i day 7GFP-. Cells which are GFP positive still express UHRF1 despite being gown in 2i conditions, enrichment of shRNA hairpins in this population of cells indicate that the factor targeted potentially plays a role in UHRF1 degradation upon resetting in 2i conditions. The experiment was performed with 3 replicates. d) MDS plot showing clustering of samples, the plot shows that 2i GFP+ samples (black circles) cluster separately from Serum/LIF day 0 (red), Serum/LIF day 7 (green) and 2i GFP- samples (dark blue). e) Plot showing the fold change (on the logarithmic scale) of each hairpin (GFP+ versus GFP-) along with the average count per million of the hairpin in the GFP+ and GFP- populations (on the logarithmic scale). The dashed blue lines represent a 2-fold increase and 2-fold decrease within the GFP+population. Hairpins enriched within the GFP+, and therefore potentially involved in the regulation of URHF1, are highlighted in the red circle. Significant candidates circled are listed in Supplementary Table 1.

To decipher the pathways and processes that regulate UHRF1 in 2i conditions, we initially set out to interrogate the process of UHRF1 degradation. Previous studies have shown that UHRF1 can be degraded by the ubiquitin-mediated proteasome in response to different biological processes, such as DNA damage [33]. Furthermore, the deubiquitinase USP7 is important in stabilizing UHRF1 protein during the cell-cycle [34]. Based on this, we probed as to whether UHRF1 loss in 2i conditions is also facilitated by the proteasome by utilizing the proteasome inhibitor MG132. mESCs were cultured in 2i conditions for a total of 10 days before MG132 was added at a final concentration of 10 μM for a total of 6 hours before cell lysates were collected. In parallel, mESCs were cultured in serum/LIF conditions in the presence of 10 μM DMSO. Western blot analysis shows the expected loss of UHRF1 in 2i cells compared to serum/LIF mESCs, however MG132 treatment of the cells rescues UHRF1 expression (Figure 1a). The Western blot for UHRF1 resulted in three bands – a higher molecular weight band that corresponds to the UHRF1-GFP fusion protein, and two further bands that correspond to full-length endogenous UHRF1 (Supplementary Figure 1A). The observation of two bands for UHRF1 has been previously reported and the slower migrating band is predicted to be a modified form of UHRF1 [35]. The rescue of UHRF1 expression levels is not through an indirect effect on Uhrf1 gene expression as qPCR analysis showed no change in the level of the Uhrf1 transcript in either 2i or 2i+MG132 cells compared to serum/LIF mESCs (Figure 1b). We thus concluded that endogenous and UHRF1-GFP loss is driven by the ubiquitin-mediated proteasome during the transition to the naïve state of pluripotency in mESCs, confirming previous observations in this experimental system [32].

### A high-throughput shRNA screen identifies factors involved in degradation of UHRF1 during the transition to naïve pluripotency

**Figure 2.**
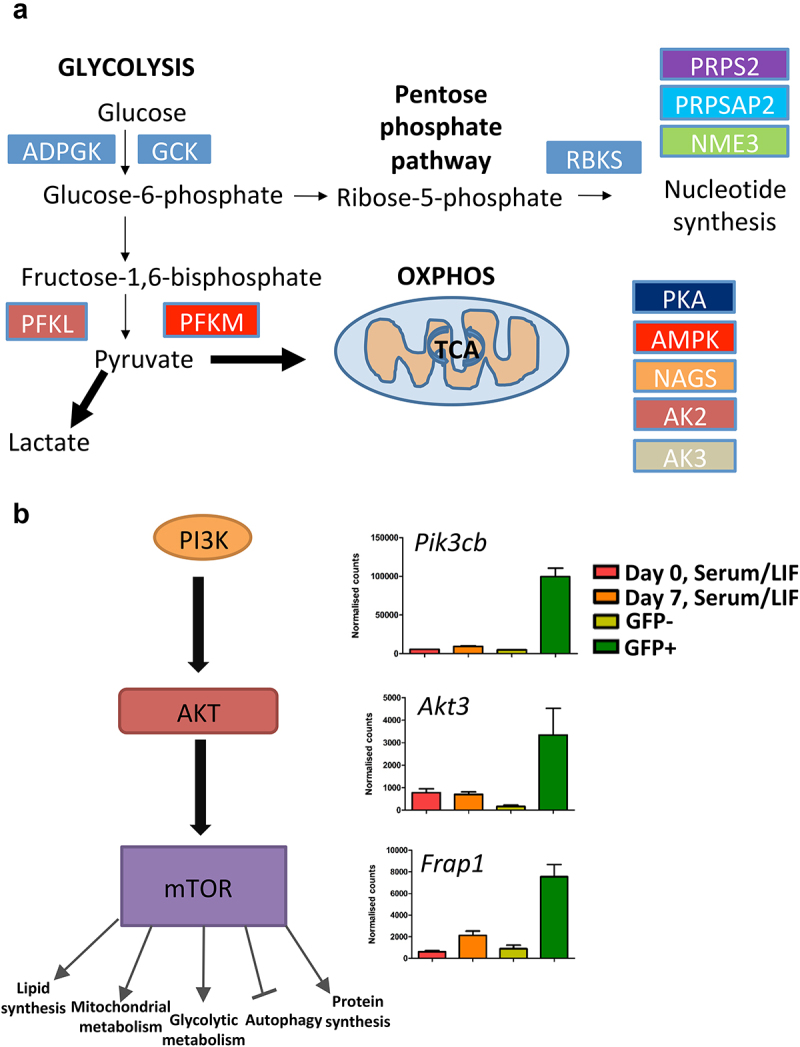
Metabolism is a key component regulating UHRF1 in mESCs. a) Schematic outlining the glycolysis, pentose phosphate pathway and oxidative phosphorylation pathways of glucose metabolism. Highlighted in coloured boxes are factors involved in the respective pathways, and that are targeted by hairpins enriched within the GFP+ population, indicating glucose metabolism is potentially a key regulator in the process of UHRF1 degradation in 2i conditions. b) Schematic of the PI3K/AKT/mTOR pathway, the bar charts show the normalized counts of three shRNAs targeting the indicated genes which form part of the pathway. The results show an enrichment of the three shRNAs in the GFP+ samples relative to the GFP+, Serum/LIF day 0 and Serum/LIF day 7 samples. Error bars represent the standard deviation from three biological replicates.

Phosphorylation of degrons is a critical step in the process of targeting a protein for degradation by the proteasome [36]. Furthermore, phosphorylation of various UHRF1 residues have been implicated in directing UHRF1 for proteasome-mediated degradation, for example, phosphorylation of S108 on UHRF1 by CK1d drives loss of UHRF1 in response to UV mediated DNA damage [33]. Given the importance of phosphorylation in the stability of UHRF1 and the fact that the induction of ground state pluripotency occurs through inhibition of signalling networks, we decided to focus our attention on the kinome to dissect the pathways regulating UHRF1 loss during the transition to naïve pluripotency. To elucidate the role of the kinome upon UHRF1 stability, we employed a high-throughput shRNA screen targeting 734 genes of the mouse kinome with a total of 3686 hairpins [37]. For the screen, we used the mESC cell line transduced with a UHRF1-GFP construct [9] and therefore used GFP expression measured by FACS as a readout for the screen. Serum/LIF cultured mESCs were transduced with the shRNA library at an MOI of 0.3 and a 500x representation of each shRNA and the cells were selected in puromycin for a total of 72 hours. Following selection, the transduced cells were grown in either serum/LIF or 2i conditions for a period of 7 days in triplicate, after which the 2i cells were sorted into GFP+ and GFP- cell populations (Figure 1c). Cells which are GFP+ in 2i conditions still express the UHRF1-GFP construct, therefore UHRF1 protein is stabilized, that is, not degraded. These UHRF1-GFP+ cells have an enrichment of shRNAs, which signal to downregulate UHRF1. Evaluation of shRNA representation in each of the cell populations by MDS plot analysis revealed that the GFP+ cluster separately to the remaining cell samples, indicating that the GFP+ cell population are characterized by a unique enrichment of hairpins (Figure 1d). A more focused pairwise analysis between the GFP+ and GFP- samples identified a total of 318 hairpins, representing 251 genes, that are enriched within the GFP+ population (logFC > 1, Benjamini–Hochberg P value <0.05) (Figure 1e – red circle, Supplementary Figure 1B and Supplementary Table 1). Out of these, 55 genes had at least 2 shRNAs present (Supplementary Figure 1C). Since 4–5 hairpins represent each gene, but not all hairpins are guaranteed to knockdown the gene, we included all 251 genes as positive candidates. Amongst the total list of genes targeted by the enriched hairpins were known interactors of UHRF1 including PKA, CKI, CKII, RIPK3 and PIM1. Recent studies have revealed the dynamic regulation and repurposing of metabolic pathways during the acquisition of naïve pluripotency [38]. Further to this, several metabolic by-products are key substrates of epigenetic regulators [39] – intrinsically linking metabolism with pluripotency and epigenetics [40,41]. We observed that several components of glucose metabolism were enriched within the list of GFP+ hits, such as GCK, PFKL and PFKM of the glycolysis pathway, RBKS, PRPS2, PRPSAP2 and NME3 of the PPP (pentose phosphate pathway) as well as several factors required for oxidative phosphorylation (Figure 2a).

Transition to ground state pluripotency involves a shift in glucose metabolism from purely glycolytic in serum/LIF mESCs to bivalent (glycolytic and oxidative phosphorylation) in naïve mESCs suggesting that glucose metabolism is a key component of naïve pluripotency [42]. Furthermore, downregulation of several components of the glycolytic pathway leads to loss of naïve pluripotency [43]. The observation that hairpins targeting factors regulating metabolism and specifically glucose metabolism are enriched within the GFP+ population confirms the success and relevance of the screen. In addition, we also observed several components of the PI3K/AKT/mTOR pathway that were targeted by enriched hairpins within the GFP+ population (Figure 2). The PI3K/AKT/mTOR pathway is a key regulator of metabolism and has been implicated in naïve cell biology in both mouse and human ESCs, and mTOR has been implicated as a regulator of the core naïve pluripotency maintenance network [44–46]. Other notable candidates include multiple hairpins of the EPH family of receptors, a large family of tyrosine kinases with roles in cytoskeletal organization, cell adhesion and morphogenesis, in particular of the nervous and lymphatic system [47].

### UHRF1 expression is rescued in validation knockdowns

**Figure 3.**
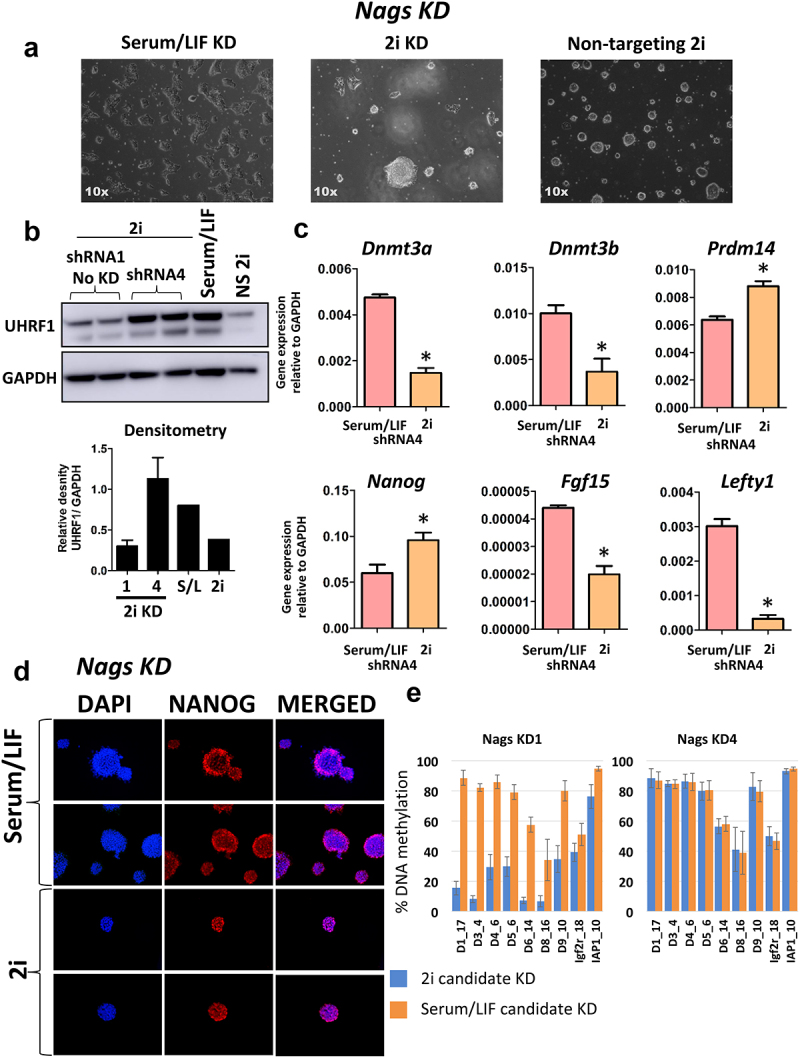
Knockdown of Nags rescues expression of UHRF1 in 2i conditions. a) Nags KD ESCs grown in 2i conditions display an altered heterogeneous morphology, data is shown for Serum/LIF KD and 2i Nags KD ESCs generated by shRNA4. b) Western blot analysis for UHRF1 upon knockdown of Nags. Analysis was performed for cell lines generated from the single shRNA producing a successful knockdown (shRNA4) and additionally shRNA1 that failed to knockdown Nags when measured by qPCR. The Western blot was performed in duplicate along with a positive control (Serum/LIF ESCs), as well as cells transduced with a non-silencing control vector grown in 2i conditions (NS 2i). Densitometry analysis was performed displaying the density of the UHRF1 band relative to the GAPDH band, error bars represent the minimum and maximum values from 2 technical replicates. The results show that knockdown of Nags prevents degradation of UHRF1 in cells cultured in 2i conditions, and therefore replicates the results of the shRNA screen. Furthermore, shRNA1 failed to impact upon UHRF1 expression. c) Six candidate genes whose expression is known to change from Serum/LIF to 2i were measured in Nags KD ESCs shRNA4, grown in both Serum/LIF and 2i conditions. Data are presented as 2− ΔCt with Gapdh serving as a loading control. Error bars represent the standard error of the mean from 3 technical replicates, * denotes P value < 0.05. d) Expression of the pluripotency marker NANOG as measured by immunofluorescence. Data is shown for Nags KD ESCs grown in both Serum/LIF and 2i. Data is shown for 2 representative images, and DAPI was used as a nuclear stain. e) DNA methylation in each KD cell line is shown in the two panels. Data is shown as average of all CpG values in each amplicon (as detailed in Supplementary Figure 6 and methods section) and error bars represent the standard deviation.

**Figure 4.**
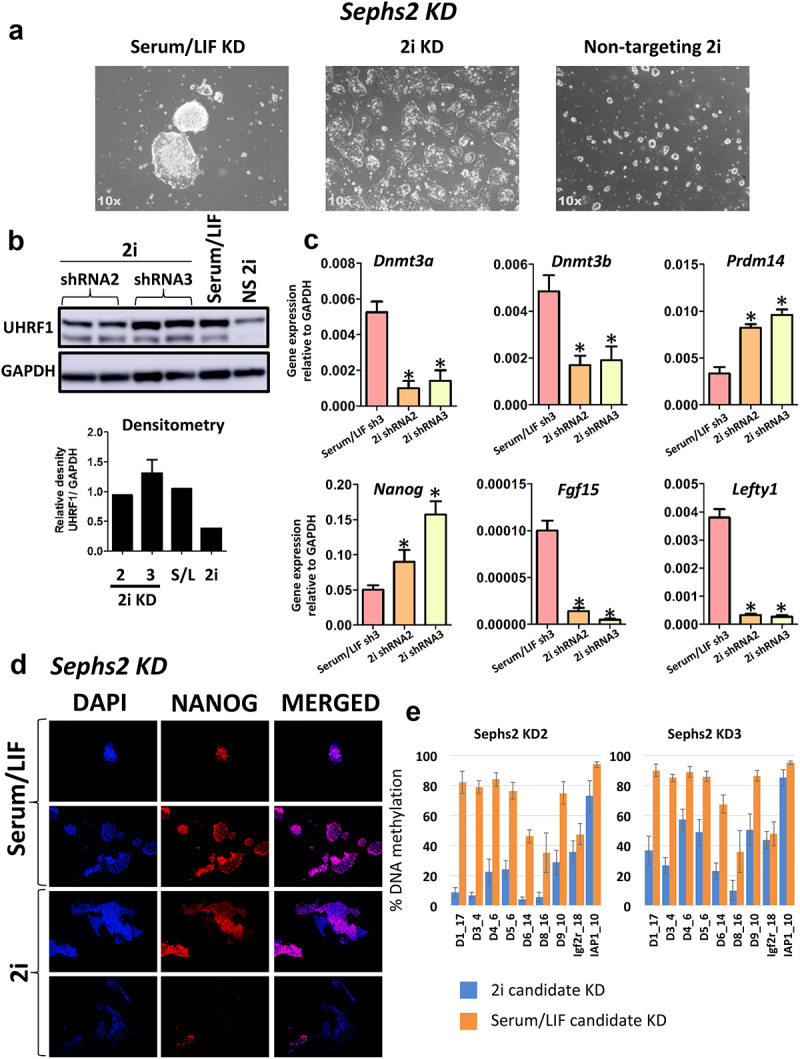
Knockdown of Sephs2 rescues expression of UHRF1 in 2i conditions. a) Sephs2 KD ESCs grown in Serum/LIF and 2i conditions display an altered morphology, data is shown for Serum/LIF and 2i Sephs2 KD ESCs generated by shRNA3. b) Western blot analysis for UHRF1 upon knockdown of Sephs2. Analysis was performed for cell lines generated from the two shRNAs producing a successful knockdown. The Western blot was performed in duplicate along with a positive control (Serum/LIF ESCs), as well as cells transduced with a non-silencing control vector grown in 2i conditions (NS 2i). Densitometry analysis was performed displaying the density of the UHRF1 band relative to the GAPDH band, error bars represent the minimum and maximum values from 2 technical replicates. The results show that knock-down of Sephs2 prevents degradation of UHRF1 in cells cultured in 2i conditions, and therefore replicates the results of the shRNA screen. c) Six candidate genes whose expression is known to change in Serum/LIF to 2i were measured in Sephs2 KD ESCs grown in both Serum/LIF and 2i conditions. Data is shown for Serum/LIF Sephs2 KD ESCs (Serum/LIF shRNA3), and 2i ESCs transduced with the 2 effective shRNA downregulating Sephs2 (shRNA2 and shRNA3). Data are presented as 2− ΔCt with Gapdh serving as a loading control. For the Serum/LIF Sephs2 KD ESCs data is shown for ESCs transduced with shRNA 3 targeting Sephs2. Error bars represent the standard error of the mean from 3 technical replicates, * denotes P value < 0.05. d) Expression of the pluripotency marker NANOG as measured by immunofluorescence. Data is shown for Sephs2 KD ESCs grown in both Serum/LIF and 2i conditions similar to Figure 3. e) DNA methylation in each KD cell line is shown in the two panels. Data is shown as average of all CpG values in each amplicon (as detailed in Supplementary Figure 6) and error bars represent the standard deviation.

**Figure 5.**
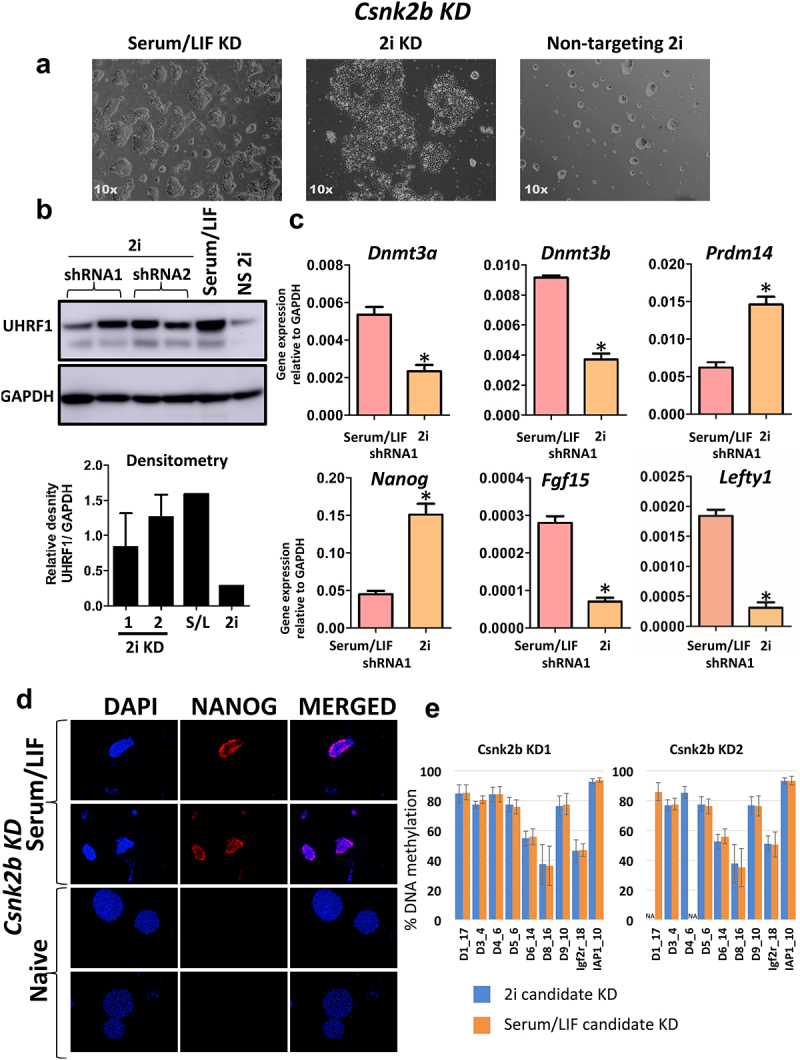
Knockdown of Csnk2b rescues expression of UHRF1 in 2i conditions. a) Csnk2b KD ESCs grown in 2i conditions display an altered morphology, with cells failing to form the domed, round colonies characteristic of 2i cells. Data is shown for Serum/LIF KD and 2i Csnk2b KD ESCs generated by shRNA1. b) Western blot analysis for UHRF1 upon knockdown of Csnk2b. Analysis was performed for cell lines generated from the two shRNAs producing a successful knockdown. The Western blot was performed in duplicate along with a positive control (Serum/LIF ESCs), as well as cells transduced with a non-silencing control vector grown in 2i conditions (NS 2i). Densitometry analysis was performed displaying the density of the UHRF1 band relative to the GAPDH band, error bars represent the minimum and maximum values from 2 technical replicates. The results show that knock-down of Csnk2b prevents degradation of UHRF1 in cells cultured in 2i conditions, and therefore replicates the results of the shRNA screen. c) Six candidate genes whose expression is known to change in Serum/LIF to 2i were measured in Csnk2b KD ESCs shRNA1, grown in both Serum/LIF and 2i conditions. Data are presented as 2− ΔCt with Gapdh serving as a loading control. Error bars represent the standard error of the mean from 3 technical replicates, * denotes P value < 0.05. d) Expression of the pluripotency marker NANOG as measured by immunofluorescence. Data is shown for Csnk2b KD ESCs grown in both Serum/LIF and 2i. Data is shown for 2 representative images, and DAPI was used as a nuclear stain. Data is for the knockdown cell line produced by shRNA1. e) DNA methylation in each KD cell line is shown in the two panels. Data is shown as average of all CpG values in each amplicon (as detailed in Supplementary Figure 6) and error bars represent the standard deviation in 3 technical replicates.

In order to confirm the accuracy of the screen, we selected four candidate genes to validate, Prpsap2, Sephs2, Csnk2b and Nags each of which are represented amongst the top enriched shRNAs within the GFP+ population, and involved in metabolic pathways (Supplementary Figure 1B). We generated cell lines, using shRNA lentiviruses, for each candidate gene. The pools of shRNAs used for validation are the same sequences as in the kinase sub-library. For Prpsap2, Sepsh2 and Csnk2b we identified 2x shRNAs that produced a knock-down of their respective conditions in both serum/LIF and 2i conditions, whereas only one such shRNA was identified targeting Nags in the two culture conditions (Supplementary Figure 2A-D). This confirms and explains the result of the shRNA screen where not all hairpins for a candidate, for example Nags, were identified in the GFP+ population. For both Prpsap2 and Sephs2 a third shRNA produced a significant knockdown of the respective genes, however it was not to the same extent as the two other successful knockdowns and so was not taken forward for further analysis. For each candidate, shRNA transduction was performed in serum/LIF cultured cells which were subsequently transitioned to the ground state and cultured in 2i media for approximately 14 days before UHRF1 protein levels were determined by Western blot. Knockdown of each of the validation targets impaired degradation of UHRF1 in mESCs grown in 2i conditions whereas cells transduced with a non-targeting shRNA resulted in the loss of UHRF1, implicating these factors in the regulation of UHRF1 and in naive pluripotency more generally (data for Nags KD – Figure 3; Sephs2 KD – Figure 4; Csnk2b KD – Figure 5; Prpsap2 – Supplementary Figure 3; Panel B Western blot in each figure shows rescue of the UHRF1 protein in each knockdown). We also performed Western blot analysis on one of the mESC cell lines transduced with an shRNA that failed to knockdown Nags, therefore acted as an additional non-targeting control (Figure 3b). Validated knockdown cell lines undergo morphological changes in 2i conditions, indicating potential cell physiological relevance, while mESCs normally form highly homogenous small, round and domed colonies. For each of the knockdowns the cells remained viable in 2i conditions and were cultured for up to 1 month.

Among the candidates we tested, Nags (N-acetylglutamate synthase) is involved in the synthesis of N-acetylglutamate (from Acetyl-CoA and Glutamate), which in mammals is an essential cofactor in the first enzymatic step of the urea cycle [48]. Nags KD ESCs populations are shown in Figure 3a, with the expected colony structure. Whilst we observed that UHRF1 expression is rescued upon knockdown of our validation candidates, we did not know whether the cells were still successfully adopting ground state pluripotency identity. To begin to address this, we selected six candidate genes that undergo well characterized expression changes in the transition to ground state pluripotency [8]. We selected Dnmt3a, Dnmt3b, Lefty1, Fgf15, which are all down-regulated in 2i, and Nanog, Prdm14 that are both up-regulated in 2i conditions (Supplementary Figure 2 E and F). Also, to probe the impact on pluripotency further we also investigated the expression of the pluripotency factor NANOG by immunofluorescence in both serum/LIF and 2i conditions, and measured alkaline phosphatase activity. Transcriptionally, Nags KD cells followed the expected transcriptional changes when transitioned from Serum/LIF to 2i culturing, and in both conditions NANOG protein levels were preserved (Figure 3c and d, non-targeting samples in Supplementary Figure 3D). Increased alkaline phosphatase (AP) activity is a marker of pluripotent stem cells [49], and so to gain an indication as to the impact of Nags KDs on the pluripotency of the mESCs, we measured AP in the cell lines by colourimetric assay. We found that AP activity was reduced in the 2i Nags KD cells, indicating impairment of naive pluripotency (Supplementary Figure 6A). Despite a 2i transcriptional state indicating transition to the ground state, albeit limited to these six marker genes, it is unclear what the significance of this reduces AP activity is. A deeper assessment of the roles of Nags in stem cell function and its relation to DNA methylation would give further answers.

Rescue of UHRF1 levels implies that DNA methylation levels would also be preserved, and would not be lost as expected during the transition to the ground state pluripotency. To test this, we have selected genomic regions known to lose substantial amounts of DNA methylation as previously described [8], and quantified DNA methylation (Supplementary Figure 6B). We also selected two genomic regions, which do not lose DNA methylation levels: an IAP (intracistronic A particle) and Igf2 (an imprinted region), also previously validated in the same study. Having these regions selected, we generated targeted bisulphite sequencing libraries and sequenced ~300 bp of each amplicon, obtaining data for several CpGs for each regions. As expected, all regions analysed validated previously published data, indicating the remarkable reproducibility of DNA methylation loss from Serum/LIF to ground state (Supplementary Figure 6B). As hypothesized, this test revealed that Nags KD prevented DNA methylation loss in the regions analysed (Figure 3e). As expected, in the samples where the shRNA was not successful in knocking down Nags (shRNA1), the methylation levels were indistinguishable from 2i levels, strengthening the direct involvement of Nags in controlling DNA methylation levels through UHRF1. Whether Nags is indirectly regulating UHRF1 degradation, mediated by other kinases, or through direct interaction is unclear.

Our next candidate, Sephs2 (selenophosphate synthetase 2), is a key enzyme in selenocysteine biosynthesis in mammals. Selenocysteine (Sec) is the 21st aminoacid in the genetic code and, unlike all other aminoacids, its biosynthesis occurs on its tRNA [50]. Sephs2 is also a selenoprotein, which means that it can autoregulate as well as control synthesis of other selenoproteins [51]. Images of Sephs2 KD populations in Serum/LIF and 2i are shown in Figure 4a, which shows that the cells didn’t maintain colony structures. Transcriptionally, the transition to the ground state occurs as expected (Figure 4c), and despite aberrant 2i morphology, NANOG protein levels are in part preserved (Figure 4d), albeit these cells have reduced AP activity (Supplementary Figure 6A). The disconnect between the lack of 2i morphology yet ground state-like gene expression of the markers in Figure 4c indicates that Sephs2 KD might affect genes linked to cytoplasmic architecture or cell-to-cell contact, but not the core pluripotency network. Interestingly, despite UHRF1 protein levels being rescued upon Sephs2 KD, DNA methylation on the target sites was still largely lost during the transition to 2i (Figure 4e), strengthening the argument that these cells transitioned to the ground state. This was unexpected and suggests that UHRF1 protein, although not degraded, is not fully functional in Sephs2 KD cells. Knockdown of Sephs2 in NIH3T3 cells resulted in significant impairment of selenoprotein synthesis, and resulted in loss of some candidate proteins tested [52]. This indicates that selenoproteins might be implicated in UHRF1 protein degradation, but also its activity, which is impaired in 2i in the absence of Sephs2. This could indicate that UHRF1 itself might be a selenoprotein.

The next candidate, Csnk2b (Casein Kinase 2 Beta), encodes the beta subunit of caseine kinase II, a broad specificity protein kinase involved in control of cellular homoeostasis [53]. Csnk2b have lost the dome-shaped colony structure in 2i (Figure 5a) but express the panel of markr genes as expected in 2i (Figure 5c), indicating that 2i-specific core network of genes are induced. While Nanog gene expression is still slightly higher in 2i as expected, NANOG protein is lost (Figure 5c and d) and these cells also have a very low AP activity compared to wild-type 2i mESCs (Supplementary Figure 6A). This could indicate that KD of Csnk2b, as a key signalling hub, affects downstream protein function, and while it rescues UHRF1 protein, it targets other proteins for degradation. RNA-seq and proteomics would clarify and delineate these possibilities, which we haven’t addressed here. We also measured levels of DNA methylation in KD cell lines in Serum/LIF and 2i conditions. Interestingly, Csnk2b KD cells, like Nags KD, do not undergo DNA methylation loss. This data shows that Nags and Csn2b KD cell lines follow the expected outcome by transitioning successfully to the ground state, while preventing UHRF1 and DNA methylation loss.

Finally, Prpsap2 (Phosphoribosyl Pyrophosphate Synthetase Associated Protein 2), is a non-catalytic associated unit of phosphoribosylpyrophosphate synthetase (PRPS), involved in the synthesis of phosphoribosyl pyrophosphate (PRPP), a primary substrate and critical control factor for de novo synthesis of purine and pyrimidine nucleotides, histidine and tryptophan, and the cofactor NAD [54]. Prpsap2 KD mESCs morphology seems to be altered (Supplementary Figure 3A) but again, the expression of all six of the test candidate genes in Prpsap2 go in the expected direction (Supplementary Figure 3C). This indicates that, at least transcriptionally, the knockdown ESCs are still transitioning to the ground state of pluripotency despite the fact that the cells still express UHRF1. We observed that Prpsap2 knockdown did not impact on NANOG in Serum/LIF conditions but resulted in reduced protein levels in 2i conditions (Supplementary Figure 3D) indicating a loss of pluripotency, despite expected transcriptional changes in 2i. We observed that Prsap2 KD resulted in reduced AP activity in 2i conditions, further indicating loss in the pluripotent capacity of these cells (Supplementary Figure 6A). Surprisingly, however, Prpsap2 KD-like Sephs2KD did not prevent DNA methylation loss despite high levels of UHRF1 levels, confirming the transition to the ground state. This indicates that UHRF1 is not fully functional, despite Prpsap2 KD transitioning to 2i with intact UHRF1, and interferes with the process of DNA methylation recruitment to the chromatin, or perhaps interferes with its enzymatic activity.

From these results, we could conclude that knockdown of all candidates tested resulted in UHRF1 protein rescue, validating the screen, but yielded varied effects on UHRF1 activity and DNA methylation maintenance. KD of all candidates reduced AP activity, indicating reduced pluripotency, and in Prpsap2 and Csnk2b KD mESCs this was associated with reduced NANOG expression. In contrast, whilst Nags and Sephs2 knockdown also resulted in reduced AP activity, NANOG expression was unaffected and further investigation is required to characterize the impact on pluripotency upon their knockdown. It is also interesting to note that the loss of NANOG following Prpsap2, Csnk2b is only evident in 2i cells, with no impact on NANOG in serum/LIF conditions. This would indicate that the role of these factors differs between the two conditions, perhaps reflective of the shift in signalling networks that mESCs undergo in 2i conditions.

### PKA signalling as a central hub in UHRF1 regulation

In addition to the validation of the screen through shRNA knockdown of candidate genes, we also validated the screen through the use of the small-molecule PKA inhibitor H-89. Inhibition of PKA was selected as several components of the heterotetramer were targeted by GFP+ enriched shRNAs, including Prkar1a, Prkar2a and Prkaca. Furthermore, regulators of PKA, such as Pkia and Pkig, were also targeted. Initially, we treated serum/LIF cells with the PKAi (final concentration of 10 μM) before transitioning the cells to 2i media for a period of 10 days (Supplementary Figure 4A). Under these conditions, PKAi treated cells transitioned to 2i forming domed colonies (Supplementary Figure 4B). Western blot analysis revealed that UHRF1 is not targeted for degradation in 2i cells grown in the presence of H-89, indicating that PKA function is required for loss of UHRF1 in the ground state (Supplementary Figure 4C). The advantage of using a small-molecule inhibitor to validate a target candidate is that it allows the inhibition after cells have transitioned to ground state pluripotency and circumvents the technical difficulties we experienced in transducing 2i cultured mESCs with shRNA constructs. Knockdowns performed in serum/LIF and subsequently transitioned to ground state could also potentially confound results by limiting the ability to differentiate between a direct effect on UHRF1 from a more general effect on the transition to naïve pluripotency and indirect effect on UHRF1 degradation. Therefore, using the inhibitor after transition sidesteps the issue of the more general effect on blocking the transition. We consequently cultured mESCs in 2i media for 10 days before adding H-89 (at a final concentration of 10 μM) for a further 10 days (Supplementary Figure 5A). Again, these cells had altered morphology from that expected of 2i cells but were also distinct from cells, which were treated with H-89 prior to the transition to the ground state pluripotency, with the presence of flat, pebble-like colonies (Supplementary Figure 5B). Expression of UHRF1 was rescued by addition of H-89 post resetting (Supplementary Figure 5C and D), indicating that PKA may play a more direct role in the regulation of UHRF1.

Next, we assayed the impact of PKAi on the acquisition of a 2i-specific gene expression profile. Interestingly, we observed that PKAi led to a failure of mESCs to downregulate Dnmt3a and Dnmt3b. Furthermore, Lefty1 and Fgf15 were upregulated upon inhibition of PKA in 2i conditions, the opposite direction to that expected. Finally, Nanog and Prdm14 were both up-regulated as expected (Supplementary Figure 4D). From these results, we could conclude that PKA inhibition results in a failure of mESCs to acquire a ground state transcriptional profile, indicating a failure of these cells to induce transition to ground state pluripotency.

In summary, our kinase screen has identified many candidate genes that play a role in regulating UHRF1 stability and activity in mouse embryonic stem cells and during transition to ground state pluripotency. Interestingly, despite the fact that knockdown of all of the genes we chose for validation rescued UHRF1 loss, not all of the knockdowns preserved DNA methylation levels, as assayed in our targeted sequencing analysis – indicating a more complex mechanism for DNA methylation inheritance than expected.

## Discussion

Here, we have conducted an shRNA kinase screen and identified 251 genes that could play a role in controlling the DNA methylation maintenance machinery. Although these candidates are involved in DNA methylation maintenance in the transition to the ground state pluripotency, they might have roles in other developmental stages, cancer or ageing. Recent evidence shows that at least in cancer cell lines, UHRF1 is regulated by the ERK pathway at the transcriptional level rather than by the proteasome [55]. However, UHRF1 regulation is complex in cancer [56], and likely the candidates we identify here will have a role in regulating UHRF1 activity in the cancer context too. This dataset, therefore, is a useful resource for the research community to further explore links between signalling pathways and DNA methylation biology, and also for a more basic understanding of the mechanism of DNA methylation inheritance.

The epistatic relationship between the genes identified in this screen remains to be established, some may be more upstream in the signalling pathway converging on UHRF1, some may be directly acting on post-translational modifications of UHRF1 to regulate its stability and activity [57]. The readout of the screen presented here is the rescue of UHRF1, i.e., preventing degradation of the protein, but what our screen revealed is that in some cases, while this is necessary in being detected as a candidate, may not be sufficient for the maintenance of the DNA methylation patterns. All of our candidates we selected and independently knocked down to validated the screen have prevented UHRF1 degradation. Interestingly, while silencing two of the candidates tested, Nags and Csnk2b, fully rescued DNA methylation loss, two of the other candidates, Prpsap2 and Sephs2, failed to preserve DNA methylation patterns despite rescuing UHRF1 loss (Figures 3–5 and Supplementary Figures 3 and 6B).

It is rather surprising that so many kinases are able to impact on UHRF1, either by modulating its activity, interaction with other proteins or proteasomal degradation. In the particular case of kinases involved in glycolysis, this indicates that the relationship between epigenetic regulation and glucose metabolism is reciprocal. This means that while epigenetic marks can control and impact on metabolism [58], activity of kinases in metabolic pathways can signal to UHRF1 protein regulation, and therefore on the DNA methylation landscape. Unpicking kinase functions and mechanisms for all candidates identified in this study appears challenging. But the mouse embryonic stem cell system, where survival of cells is not reliant on DNA methylation (knockout cells lacking all methyltransferases are viable [59]) is a perfect system to delineate the mechanisms of UHRF1 regulation. This is not the case for differentiated cells, which require DNA methylation [60].

Inhibition of PKA using a small molecule inhibitor prevented UHRF1 degradation but had variable effects on DNA methylation levels, although most of the sites analysed have maintained DNA methylation. Loss of DNA methylation is thought to be a concerted regulation of two pathways, degradation of UHRF1 and global reduction of H3K9me2 [9], therefore, there is a possibility that Prsap2 and Sephs2 interfere with the lysine 9 methyltransferase activity or stability of the protein complexes they have been previously shown to be a part of [61]. The reliance on H3K9me2 for DNA methylation maintenance seems a bit more complex and nuanced since a catalytic mutation of G9a, the enzyme catalysing H3K9me2 deposition, resulted in a very small, albeit significant, reduction of global DNA methylation levels in mouse embryonic stem cells (less than 10% compared to the wild-type mESCs) while full G9a knockout in the same cells led to ~4 fold more loss of global DNA methylation [62], supporting the role of other H3K9 methyltransferases needed for the catalytic activity and dependence of the stability of the protein complexes on G9a. An alternative explanation might lie in the involvement of Prpsap2 and Prps2 (also a resulting candidate in our screen) in NAD production [63]. HDACs and sirtuins are NAD dependent proteins [64] and previous studies have identified that SIRT1 and DNMT1 can strongly bind to chromatin under oxidative stress, without changing the expression levels of DNMT1 [65]. There are no reports on the interaction of SIRT1 with DNMT1 in the transition to mouse ground state pluripotency but it is possible that impaired binding of DNMT1 to the chromatin is the reason DNA methylation is not maintained in Prsap2 KD cells despite the presence of UHRF1. Overall, the role of Prsap2 and Sephs2 in interfering with H3K9 methyltransferases or an alternative mechanism to inhibit DNA methylation maintenance remains to be determined. The complete rescue of the DNA methylation upon Nags and Csnk2b knockdown is notable. There is no documented link between Nags or N-acetylglutamate and histone PTM (Post-translational modification) or DNA methylation machinery, and at this stage it is unclear how this gene might regulate DNA methylation maintenance. Regarding Csnk2b, there is precedence in regulation of UHRF1 by Caseine Kinase 1 delta [33], and we hypothesize that the most likely mechanism of UHRF1 stabilization by Csnk2b is though a PTM. Generally, an interesting next step in following up the candidates from our screen, in particular Nags and Csnk2b, is to analyse UHRF1 and the resulting PTMs which might target UHRF1 for degradation by the proteasome. Another factor directly involved in the regulation of UHRF1 by targeting UHRF1 for degradation in mouse embryonic stem cells is Pramel7 [32]. Since our study is a kinase screen, we did not identify Pramel7 but it is very likely that our candidates either regulate Pramel7 levels, or more likely, interfere with PRAMEL7 interaction with UHRF1, which remains to be determined. Regarding the candidates we have identified in this screen, there is a possibility that some of them could reverse the inhibition by 2i and cells that have not transitioned to ground state might enrich in the GFP+ population. While this is a possibility, our validation data argues against this, where in some cases DNA methylation was still lost when in 2i state despite intact UHRF1 protein. Even PKA, which when inhibited induces differentiation in stem cells, seems to be involved in signalling and controlling UHRF1 degradation in the ground state. Our data is also supporting the concept that DNA methylation and pluripotency might not be intimately linked [8,66], and stem cells can transition to the naive state even in the presence of DNA methylation. The reason perhaps such a screen could work is because the presence of DNA methylation may not be relevant in naive pluripotency, and allows detection of functional relationships between signalling events and DNA methylation maintenance without impacting on the survival of cells. Nevertheless, robust DNA methylation machinery is essential for differentiation [67].

Regarding morphology of mESCs in Serum/LIF and 2i conditions, examining all the KD cells we have generated throughout the validation process indicates that there is little relationship between gene expression status and colony morphology. Although many of the knockdowns showed altered morphology in 2i, their gene expression pattern (the six marker genes) was 2i-like. Conversely, gene expression of PKAi cells transitioned to 2i had colony morphology similar to 2i cells (Supplementary Figure 4B) but our panel of six genes indicated differentiation. Therefore, one cannot use morphology to infer ground state transition of cells, which is commonly done in this experimental system.

In conclusion, we are providing a platform for the research community to further explore fundamental processes in mammalian development and disease, linking to DNA methylation biology. Loss of DNA methylation is very common in cancer and there might be pertinent parallels between the mechanism of DNA methylation loss in embryonic stem cells and the process of cellular transformation. Preventing global methylation loss might therefore be a viable cancer prevention avenue.

## Materials and methods

### Culture of mESCs

E14 ESCs in the Serum/LIF state were cultured without feeders in serum containing media (DMEM 4500 mg/l glucose, 4 mM L-glutamine, 110 mg/l sodium pyruvate, 15% foetal bovine serum, 1 U/ml penicillin, 1 μg/ml streptomycin, 0.1 mM nonessential amino acids, 50 μM β-mercaptoethanol and 10^3^ U/ml ESGRO LIF). E14 ESCs in the 2i state were cultured in the 2i media containing serum-free N2B27 – [DMEM/F12, Neurobasal, N2, B27], 10^3^ U/ml ESGRO LIF, PD0325901 [1 μm] and CHIR99021 [3 μM].

### shRNA library transduction

To perform the screen we obtained the TRC lentiviral mouse kinase shRNA library from the Broad Institute. For the screen, we obtained a mouse E14 ESC cell line expressing a UHRF1-GFP transgene (a kind gift from Prof Wolf Reik Lab), thereby allowing us to indirectly detect UHRF1 levels through GFP fluorescence (von Meyenn et al.,2016). Serum/LIF mESCs were transduced with the shRNA library at an M.O.I of 0.3 and a representation of 500x for each shRNA hairpin present in the library. Transduction was carried out in the presence of polybrene at a final concentration of 8 μg/ml and cells were transduced for a period of 24 hours. Following transduction, the cell media was removed and the cells were washed 2x in PBS before fresh media was added to the cells. Twenty-four hours later cells that had been successfully transduced were selected for a period of 72 hours in the presence of puromycin at a final concentration of 1.5 μg/ml.

### shRNA screen

Following selection, the cells were propagated in Serum/LIF media before being split into two – with one-half maintained in Serum/LIF media and the other half of cells in 2i media. At this point, cells were also collected for subsequent DNA extraction and sequencing – with these samples representing the diversity of the library at the start of the screen (Sample – Serum/LIF day 0). After 7 days, the cells cultured in Serum/LIF media were collected in triplicate (sample – Serum/LIF day 7), whilst the cells cultured in 2i media were then sorted by FACS based on GFP expression (GFP+ and GFP-). At each step, at least 1.8 × 10^6^ cells were collected for each sample to ensure a theoretical representation of 500x for each shRNA.

### shRNA screen: DNA extraction and PCR amplification

Following collection of cells DNA was extracted using the DNeasy blood and tissue kit (Qiagen) using the standard procedure described in the handbook. For PCR amplification of the library 2 μg of DNA for each sample was used, with the DNA split into four 500 ng reactions. A one-step PCR approach was used with P5 and P7 primers. The P5 and P7 primer were comprised of the P5/P7 flow-cell attachment sequence, the Illumina sequencing primer and the vector-binding sequence (primer sequence in shown in Supplementary Table 2). In addition, to avoid the issue of reduced sequence diversity faced by amplicon libraries, PCR was performed with a mix of P5 primers containing a stagger region of different length. The PCR reaction was set-up on ice as follows: 10 μl ExTaq 10x reaction buffer (Takara), 8 μl dNTPs (10 mM), 0.5 μl P5 primer (100 μM), 1.5 μl ExTaq polymerase (Takara), 10 μl P7 primer (5 μM) and the reaction mix was made up to 100 μl with water. The PCR cycling parameters were as follows: 95°C 1 minute, 28 cycles (95°C 30 seconds, 53°C 30 seconds, 72°C 30 seconds), 72°C 10 minutes. The PCR product was then purified using the AMPure purification system (Beckmancoulter) ready for sequencing.

### Library sequencing and shRNA screen data analysis

Sequencing was carried out on the Illumina NextSeq 500 platform using the mid-output (~400 × 10^6^ reads). Sequencing was carried out by the Queen Mary University of London Genome Centre. Raw data and count table were deposited at GEO with the accession number GSE158453. Alignment of sequencing data was performed using Bowtie2 under standard alignment parameters. Following alignment, a count table was generated using the following Python script (https://dharmacon.horizondiscovery.com/uploadedFiles/Resources/lentiviral-pooled-librarybioinfomatic-analysis-protocol.pdf). The count table was then imported into R and differential representation analysis was performed using the package EdgeR and using the exactTest function.

### Real-time PCR

Real-time analysis of gene expression was carried out using SYBR green chemistry. Expression of target genes were measured relative to the housekeeping gene Gapdh. The relative expression of the target genes was calculated using the 2− ΔCt method: 2− ΔCt (target gene) = 2 – [Ct (target gene) – Ct (average of housekeeping genes)].

### Western blotting

The 10 μg of protein was loaded onto a 4–12% Bis-Tris gel (NuPAGE) and run with 1 X MOPS SDS running buffer (NuPAGE). The denatured proteins were transferred to a PVDF membrane, which was subsequently blocked in 5% milk. Primary antibody incubation was carried out overnight with the following antibodies and concentration – UHRF1 1:1000 (Merck – MABE308), GFP 1:1000 (Abcam – ab290) and GAPDH 1:2500 (Cell Signalling Technology – 2118s). Following primary incubation, the membrane was washed 4x with PBST (PBS+0.1% Tween20) before incubation with the appropriate secondary antibody for 40 minutes. For UHRF1, anti-mouse HRP antibody (Merck – GENA934) was used, and for GFP and GAPDH anti-rabbit HRP antibody (Merck – GENA934) at a dilution of 1:5000. Following secondary antibody incubation, the membrane was washed 4 X in PBST before a final wash in PBS. Detection of target protein levels was carried out by chemiluminescence using the SuperSignal™ West Pico PLUSchemilumiescent substrate (ThermoFisher Scientific) and the blot was imaged on the Amersham imager 600.

### Immunofluorescence

Cells were grown in 12-well plates on cover slips before the media was removed and the cells washed 2x with PBS. The cells were then fixed with 4% paraformaldehyde for 15 minutes at room temperature, after which the cells were washed 2x in PBS and permeabilised with 0.2% Triton X100 in PBS for 5 minutes at room temperature. The cells were washed 2x in PBS and subsequently blocked with 1% FCS/PBS for 30 minutes after which the Nanog primary antibody was added to the cells in a 1:500 dilution in 1% FCS/PBS (Abcam – ab150084) and incubated for 1 hour at room temperature. Following this the cells were washed 3x in PBS and were then incubated in the presence of the secondary antibody Anti-Rabbit Alexa Fluor 594 (Abcam – ab150084) diluted 1:500 in 1%FCS/PBS. The cells were then washed a further 3x in PBS before being stained with DAPI diluted 1:500 in PBS, after which the cover slips were mounted onto a glass slide for imaging.

### Alkaline phosphatase activity

Alkaline phosphatase activity was measured by colourimetric assay using the Amplite™ colourimetric Alkaline Phosphatase Assay Kit. Cells were cultured in 2i media for at least 10 days, after which 10,000 cells for each cell line/treatment were seeded onto a flat-bottomed 96 well plate and cultured for 6 hours, before which the standard protocol described in the handbook was followed.

Absorbance was measured at 400 nm wavelength using the Optima plate reader.

### Targeted bisulphite sequencing

Bisulphite PCR primers were designed against an *in silico* bisulphite converted reference sequence, and universal Illumina adapter sequences were added to the 5’ end of each primer (Supplementary Table 3). Cells were dissociated to single cells using Accutase and DNA was isolated from pelleted cells using the PureLink Genomic DNA mini kit (ThermoFisher Scientific). Bisulphite conversion of DNA was carried out using the EZ DNA methylation lightning kit (Zymo), following the manufacturer’s instructions. The modified DNA was amplified using the loci specific bisulphite PCR primers and HotStar Taq DNA Polymerase (Qiagen). The PCR conditions were as follows: 95°C for 15 min; 94°C for 30 seconds; 56°C for 30 seconds; 72°C for 1 min; Repeat steps 2–4 29X; 72°C for 10 min; Hold 12°C. PCR products were purified using SPRI beads (Agencourt AMPure XP, Beckman Coulter). Amplicons were PCR amplified with eight cycles using a universal Illumina forward primer and an indexed reverse primer and quantified with the Kapa Library quantification kit for Illumina (Roche). Amplicons from a single sample were pooled and sequencing was performed on an Illumina MiSeq with 150 bp paired-end reads, using v3 chemistry, at Barts and the London Genome Centre (London, UK). For the analysis, paired end sequences were quality checked trimmed and mapped to a custom genome with Bismark (v0.22.1) followed by extraction of methylation calls. The pipeline and code is available in the Supplementary material.

## Supplementary Material

Supplemental Material

## Highlights

- Two hundred and fifty-one genes identified which regulate UHRF1 stability in the transition to mouse ground state
- All candidates we tested prevented UHRF1 degradation when candidates were individually silenced
- Inhibition of Nags and Csnk2b rescued both UHRF1 protein levels and DNA methylation. Inhibition of Prpsap2 and Sephs2 did not rescue DNA methylation loss, despite UHRF1 presence; PKA inhibition had pleiotropic effects

## References

[1] Baylin SB, Jones PA. A decade of exploring the cancer epigenome - biological and translational implications. Nat Rev Cancer. 2011;11(10):726–734. 21941284 10.1038/nrc3130 PMC3307543

[2] Jones PA. Functions of DNA methylation: Islands, start sites, gene bodies and beyond. Nat Rev Genet. 2012;13(7):484–492. 22641018 10.1038/nrg3230

[3] Edwards JR, Yarychkivska O, Boulard M, et al. DNA methylation and DNA methyltransferases. Epigenetics Chromatin. 2017;10(1):23. 28503201 10.1186/s13072-017-0130-8 PMC5422929

[4] Bostick M, Kim JK, Esteve PO, et al. UHRF1 plays a role in maintaining DNA methylation in mammalian cells. Science. 2007;317(5845):1760–1764. 17673620 10.1126/science.1147939

[5] Rothbart SB, Krajewski K, Nady N, et al. Association of UHRF1 with methylated H3K9 directs the maintenance of DNA methylation. Nat Struct Mol Biol. 2012;19(11):1155–1160. 23022729 10.1038/nsmb.2391 PMC3492551

[6] Sharif J, Muto M, Takebayashi S, et al. The SRA protein Np95 mediates epigenetic inheritance by recruiting Dnmt1 to methylated DNA. Nature. 2007;450(7171):908–912. 17994007 10.1038/nature06397

[7] Seisenberger S, Peat JR, Hore TA, et al. Reprogramming DNA methylation in the mammalian life cycle: building and breaking epigenetic barriers. Philos Trans R Soc Lond B Biol Sci. 2013;368(1609):20110330. 23166394 10.1098/rstb.2011.0330 PMC3539359

[8] Ficz G, Hore TA, Santos F, et al. FGF signaling inhibition in ESCs drives rapid genome-wide demethylation to the epigenetic ground state of pluripotency. Cell Stem Cell. 2013;13(3):351–359. 23850245 10.1016/j.stem.2013.06.004 PMC3765959

[9] von Meyenn F, Iurlaro M, Habibi E, et al. Impairment of DNA methylation maintenance is the main cause of global demethylation in naive embryonic stem cells. Mol Cell. 2016;62(6):848–861. 27237052 10.1016/j.molcel.2016.04.025 PMC4914828

[10] Evans MJ, Kaufman MH. Establishment in culture of pluripotential cells from mouse embryos. Nature. 1981;292(5819):154–156. 7242681 10.1038/292154a0

[11] Martin GR. Isolation of a pluripotent cell line from early mouse embryos cultured in medium conditioned by teratocarcinoma stem cells. Proc Natl Acad Sci U S A. 1981;78(12):7634–7638. 6950406 10.1073/pnas.78.12.7634 PMC349323

[12] Bradley A, Evans M, Kaufman MH, et al. Formation of germ-line chimaeras from embryo-derived teratocarcinoma cell lines. Nature. 1984;309(5965):255–256. 6717601 10.1038/309255a0

[13] Martello G, Smith A. The nature of embryonic stem cells. Annu Rev Cell Dev Biol. 2014;30(1):647–675. 25288119 10.1146/annurev-cellbio-100913-013116

[14] Smith AG, Hooper ML. Buffalo rat liver cells produce a diffusible activity which inhibits the differentiation of murine embryonal carcinoma and embryonic stem cells. Dev Biol. 1987;121(1):1–9. 3569655 10.1016/0012-1606(87)90132-1

[15] Smith AG, Heath JK, Donaldson DD, et al. Inhibition of pluripotential embryonic stem cell differentiation by purified polypeptides. Nature. 1988;336(6200):688–690. 3143917 10.1038/336688a0

[16] Williams RL, Hilton DJ, Pease S, et al. Myeloid leukaemia inhibitory factor maintains the developmental potential of embryonic stem cells. Nature. 1988;336(6200):684–687. 3143916 10.1038/336684a0

[17] Nichols J, Evans EP, Smith AG. Establishment of germ-line-competent embryonic stem (ES) cells using differentiation inhibiting activity. Development. 1990;110(4):1341–1348. 2129226 10.1242/dev.110.4.1341

[18] Ying QL, Nichols J, Chambers I, et al. BMP induction of Id proteins suppresses differentiation and sustains embryonic stem cell self-renewal in collaboration with STAT3. Cell. 2003;115(3):281–292. 14636556 10.1016/s0092-8674(03)00847-x

[19] Azuara V, Perry P, Sauer S, et al. Chromatin signatures of pluripotent cell lines. Nat Cell Biol. 2006;8(5):532–538. 16570078 10.1038/ncb1403

[20] Bernstein BE, Mikkelsen TS, Xie X, et al. A bivalent chromatin structure marks key developmental genes in embryonic stem cells. Cell. 2006;125(2):315–326. 16630819 10.1016/j.cell.2006.02.041

[21] Chambers I, Silva J, Colby D, et al. Nanog safeguards pluripotency and mediates germline development. Nature. 2007;450(7173):1230–1234. 18097409 10.1038/nature06403

[22] Hayashi K, de Sousa Lopes SMC, Tang F, et al. Dynamic equilibrium and heterogeneity of mouse pluripotent stem cells with distinct functional and epigenetic states. Cell Stem Cell. 2008;3(4):391–401. 18940731 10.1016/j.stem.2008.07.027 PMC3847852

[23] Toyooka Y, Shimosato D, Murakami K, et al. Identification and characterization of subpopulations in undifferentiated ES cell culture. Development. 2008;135(5):909–918. 18263842 10.1242/dev.017400

[24] Niwa H, Ogawa K, Shimosato D, et al. A parallel circuit of LIF signalling pathways maintains pluripotency of mouse ES cells. Nature. 2009;460(7251):118–122. 19571885 10.1038/nature08113

[25] Smith ZD, Chan MM, Humm KC, et al. DNA methylation dynamics of the human preimplantation embryo. Nature. 2014;511(7511):611–615. 25079558 10.1038/nature13581 PMC4178976

[26] Guo H, Zhu P, Yan L, et al. The DNA methylation landscape of human early embryos. Nature. 2014;511(7511):606–610. 25079557 10.1038/nature13544

[27] Kinoshita M, Smith A. Pluripotency Deconstructed. Dev Growth Differ. 2018;60(1):44–52. 29359419 10.1111/dgd.12419

[28] Ying QL, Wray J, Nichols J, et al. The ground state of embryonic stem cell self-renewal. Nature. 2008;453(7194):519–523. 18497825 10.1038/nature06968 PMC5328678

[29] Wray J, Kalkan T, Gomez-Lopez S, et al. Inhibition of glycogen synthase kinase-3 alleviates Tcf3 repression of the pluripotency network and increases embryonic stem cell resistance to differentiation. Nat Cell Biol. 2011;13(7):838–845. 21685889 10.1038/ncb2267 PMC3160487

[30] Yamaji M, Ueda J, Hayashi K, et al. PRDM14 ensures naive pluripotency through dual regulation of signaling and epigenetic pathways in mouse embryonic stem cells. Cell Stem Cell. 2013;12(3):368–382. 23333148 10.1016/j.stem.2012.12.012

[31] Leitch HG, McEwen KR, Turp A, et al. Naive pluripotency is associated with global DNA hypomethylation. Nat Struct Mol Biol. 2013;20(3):311–316. 23416945 10.1038/nsmb.2510 PMC3591483

[32] Graf U, Casanova EA, Wyck S, et al. Pramel7 mediates ground-state pluripotency through prote-asomal-epigenetic combined pathways. Nat Cell Biol. 2017;19(7):763–773. 28604677 10.1038/ncb3554

[33] Chen H, Ma H, Inuzuka H, et al. DNA damage regulates UHRF1 stability via the SCF(beta-TrCP) E3 ligase. Mol Cell Biol. 2013;33(6):1139–1148. 23297342 10.1128/MCB.01191-12 PMC3592027

[34] Ma H, Chen H, Guo X, et al. M phase phosphorylation of the epigenetic regulator UHRF1 regulates its physical association with the deubiquitylase USP7 and stability. Proc Natl Acad Sci U S A. 2012;109(13):4828–4833. 22411829 10.1073/pnas.1116349109 PMC3323953

[35] Guan D, Factor D, Liu Y, et al. The epigenetic regulator UHRF1 promotes ubiquitination-mediated degradation of the tumor-suppressor protein promyelocytic leukemia protein. Oncogene. 2013;32(33):3819–3828. 22945642 10.1038/onc.2012.406 PMC3578017

[36] Schrader EK, Harstad KG, Matouschek A. Targeting proteins for degradation. Nat Chem Biol. 2009;5(11):815–822. 19841631 10.1038/nchembio.250 PMC4228941

[37] Sakurai K, Talukdar I, Patil VS, et al. Kinome-wide functional analysis highlights the role of cytoskeletal remodeling in somatic cell reprogramming. Cell Stem Cell. 2014;14(4):523–534. 24702998 10.1016/j.stem.2014.03.001 PMC4071169

[38] Mathieu J, Ruohola-Baker H. Metabolic remodeling during the loss and acquisition of pluripotency. Development. 2017;144(4):541–551. 28196802 10.1242/dev.128389 PMC5312031

[39] Wong CC, Qian Y, Yu J. Interplay between epigenetics and metabolism in oncogenesis: mechanisms and therapeutic approaches. Oncogene. 2017;36(24):3359–3374. 28092669 10.1038/onc.2016.485 PMC5485177

[40] Dahan P, Lu V, Nguyen RMT, et al. Metabolism in pluripotency: both driver and passenger? J Biol Chem. 2019;294(14):5420–5429. 29463682 10.1074/jbc.TM117.000832 PMC6462533

[41] Ryall JG, Cliff T, Dalton S, et al. Metabolic reprogramming of stem cell epigenetics. Cell Stem Cell. 2015;17(6):651–662. 26637942 10.1016/j.stem.2015.11.012 PMC4672395

[42] Teslaa T, Teitell MA. Pluripotent stem cell energy metabolism: an update. EMBO J. 2015;34(2):138–153. 25476451 10.15252/embj.201490446 PMC4337063

[43] Sun Z, Zhu M, Lv P, et al. The long noncoding RNA Lncenc1 maintains naive states of mouse ESCs by promoting the glycolysis pathway. Stem Cell Reports. 2018;11(3):741–755. 30174313 10.1016/j.stemcr.2018.08.001 PMC6135739

[44] Yu JS, Cui W. Proliferation, survival and metabolism: the role of PI3K/AKT/mTOR signalling in pluripotency and cell fate determination. Development. 2016;143(17):3050–3060. 27578176 10.1242/dev.137075

[45] Singh AM, Bechard M, Smith K, et al. Reconciling the different roles of Gsk3beta in “naive” and “primed” pluripotent stem cells. Cell Cycle. 2012;11(16):2991–2996. 22825252 10.4161/cc.21110 PMC3442909

[46] Li M, Yu JSL, Tilgner K, et al. Genome-wide CRISPR-KO screen uncovers mTORC1-mediated Gsk3 regulation in naive pluripotency maintenance and dissolution. Cell Rep. 2018;24(2):489–502. 29996108 10.1016/j.celrep.2018.06.027 PMC6057492

[47] Pasquale EB. Eph receptor signalling casts a wide net on cell behaviour. Nat Rev Mol Cell Biol. 2005;6(6):462–475. 15928710 10.1038/nrm1662

[48] Caldovic L, Tuchman M. N-acetylglutamate and its changing role through evolution. Biochem J. 2003;372(Pt 2):279–290. 12633501 10.1042/BJ20030002 PMC1223426

[49] Stefkova K, Prochazkova J, Pachernik J. Alkaline phosphatase in stem cells. Stem Cells Int. 2015;2015:628368. 25767512 10.1155/2015/628368 PMC4342173

[50] Lee BJ, Worland PJ, Davis JN, et al. Identification of a selenocysteyl-tRNA(Ser) in mammalian cells that recognizes the nonsense codon, UGA. J Biol Chem. 1989;264(17):9724–9727. 2498338

[51] Guimaraes MJ, Peterson D, Vicari A, et al. Identification of a novel selD homolog from eukaryotes, bacteria, and archaea: is there an autoregulatory mechanism in selenocysteine metabolism? Proc Natl Acad Sci U S A. 1996;93(26):15086–15091. 8986768 10.1073/pnas.93.26.15086 PMC26360

[52] Xu XM, Carlson BA, Irons R, et al. Selenophosphate synthetase 2 is essential for selenoprotein biosynthesis. Biochem J. 2007;404(1):115–120. 17346238 10.1042/BJ20070165 PMC1868833

[53] Meggio F, Pinna LA. One-thousand-and-one substrates of protein kinase CK2? FASEB J. 2003;17(3):349–368. 12631575 10.1096/fj.02-0473rev

[54] Katashima R, Iwahana H, Fujimura M, et al. Assignment of the human phosphoribosylpyrophosphate synthetase-associated protein 41 gene (PRPSAP2) to 17p11.2-p12. Genomics. 1998;54(1):180–181. 9806849 10.1006/geno.1998.5432

[55] Li J, Wang R, Hu X, et al. Activated MEK/ERK pathway drives widespread and coordinated overexpression of UHRF1 and DNMT1 in cancer cells. Sci Rep. 2019;9(1):907. 30696879 10.1038/s41598-018-37258-3 PMC6351616

[56] Kong X, Chen J, Xie W, et al. Defining UHRF1 domains that support maintenance of human colon cancer DNA methylation and oncogenic properties. Cancer Cell. 2019;35(4):633–48 e7. 30956060 10.1016/j.ccell.2019.03.003 PMC6521721

[57] Xie S, Qian C. The growing complexity of UHRF1-mediated maintenance DNA methylation. Genes (Basel). 2018;9(12):600. 10.3390/genes9120600 PMC6316679 30513966

[58] Tobi EW, Slieker RC, Luijk R, et al. DNA methylation as a mediator of the association between prenatal adversity and risk factors for metabolic disease in adulthood. Sci Adv. 2018;4(1):eaao4364. 29399631 10.1126/sciadv.aao4364 PMC5792223

[59] Tsumura A, Hayakawa T, Kumaki Y, et al. Maintenance of self-renewal ability of mouse embryonic stem cells in the absence of DNA methyltransferases Dnmt1, Dnmt3a and Dnmt3b. Genes Cells. 2006;11(7):805–814. 16824199 10.1111/j.1365-2443.2006.00984.x

[60] Liao J, Karnik R, Gu H, et al. Targeted disruption of DNMT1, DNMT3A and DNMT3B in human embryonic stem cells. Nat Genet. 2015;47(5):469–478. 25822089 10.1038/ng.3258 PMC4414868

[61] Fritsch L, Robin P, Mathieu JR, et al. A subset of the histone H3 lysine 9 methyltransferases Suv39h1, G9a, GLP, and SETDB1 participate in a multimeric complex. Mol Cell. 2010;37(1):46–56. 20129054 10.1016/j.molcel.2009.12.017

[62] Zhang T, Termanis A, Ozkan B, et al. G9a/GLP complex maintains imprinted DNA methylation in embryonic stem cells. Cell Rep. 2016;15(1):77–85. 27052169 10.1016/j.celrep.2016.03.007 PMC4826439

[63] Qian X, Li X, Tan L, et al. Conversion of PRPS hexamer to monomer by AMPK-mediated phosphorylation inhibits nucleotide synthesis in response to energy stress. Cancer Discov. 2018;8(1):94–107. 29074724 10.1158/2159-8290.CD-17-0712 PMC5760453

[64] Schemies J, Uciechowska U, Sippl W, et al. NAD(+) -dependent histone deacetylases (sirtuins) as novel therapeutic targets. Med Res Rev. 2010;30(6):861–889. 19824050 10.1002/med.20178

[65] O’Hagan HM, Wang W, Sen S, et al. Oxidative damage targets complexes containing DNA methyltransferases, SIRT1, and polycomb members to promoter CpG Islands. Cancer Cell. 2011;20(5):606–619. 22094255 10.1016/j.ccr.2011.09.012 PMC3220885

[66] Lynch CJ, Bernad R, Martinez-Val A, et al. Global hyperactivation of enhancers stabilizes human and mouse naive pluripotency through inhibition of CDK8/19 mediator kinases. Nat Cell Biol. 2020;22(10):1223–1238. 32989249 10.1038/s41556-020-0573-1

[67] Smith ZD, Meer A. DNA methylation: roles in mammalian development. Nat Rev Genet. 2013;14(3):204–220. 23400093 10.1038/nrg3354

