# Supplementary material for: An shRNA kinase screen identifies regulators of UHRF1 stability and activity in mouse embryonic stem cells
Source: Epigenetics. 2022 Mar 24;17(12):1590–607. doi: 10.1080/15592294.2022.2044126 (PMC9621053; doi:10.1080/15592294.2022.2044126)
Supplement: Supplemental Material [file KEPI_A_2044126_SM0147.zip › Rushton_supplementary/Sequences_pooledshRNAs.docx]

**Clone ID and mature antisense sequence**

**Gene set: TRC Nags shRNA**

TRCN0000076073 TAAGGTTACCTTAGGTAGAGC

TRCN0000076074 ATCTTCTGACTGTTATTCCGC

TRCN0000076075 TAGCGGCTGTAATGACTGCGG

TRCN0000076076 TTGGCATGATTGACTAGTTCG

TRCN0000076077 ATAGATCGAGTGCAACCGAGG

**Gene set: TRC Sephs2 shRNA**

TRCN0000075758 AATAGCCTCCTCTTGCTTTGG

TRCN0000075759 TAGAACTTCAATAACGCGAGG

TRCN0000075760 TATTGGCAGATTATGTATGAC

TRCN0000075761 AGAACTTCAATAACGCGAGGC

TRCN0000075762 TAAAGGTTTGGTTAATACCAG

**Gene set: TRC Csnk2b shRNA**

TRCN0000025054 TTTCCAACATTTGTGCGATGC

TRCN0000025055 AAGAATTCATTACCACGGAGC

TRCN0000025056 TTCCAGGTCTAAGATCATGTC

TRCN0000025057 ATGGATCTTGAAACCATAGAG

TRCN0000025058 ATGCACCATGAAGAGCATGTG

**Gene set: TRC Prpsap2 shRNA**

TRCN0000075708 TAACTTCTTTCATTTCAGAGG

TRCN0000075709 AATGTCATCCACAATGATGGC

TRCN0000075710 TTGCACATCATAGAAGCCAGC

TRCN0000075711 TTTGCACATCATAGAAGCCAG

TRCN0000075712 TAATGTCATCCACAATGATGG
