# Supplementary material for: An shRNA kinase screen identifies regulators of UHRF1 stability and activity in mouse embryonic stem cells
Source: Epigenetics. 2022 Mar 24;17(12):1590–607. doi: 10.1080/15592294.2022.2044126 (PMC9621053; doi:10.1080/15592294.2022.2044126)
Supplement: Supplemental Material [file KEPI_A_2044126_SM0147.zip › Rushton_supplementary/SupplementaryTables2_3.docx]

| **Primer name** | **Sequence 5´ to 3´** |
| --- | --- |
| P5 | AATGATACGGCGACCACCGAGATCTACACTCTTTCCCTACACGACGCTCTTCCGATCTTCTTGTGGAAAGGACGA |
| P5 – A stagger | AATGATACGGCGACCACCGAGATCTACACTCTTTCCCTACACGACGCTCTTCCGATCTATCTTGTGGAAAGGACGA |
| P5 – GA stagger | AATGATACGGCGACCACCGAGATCTACACTCTTTCCCTACACGACGCTCTTCCGATCTGATCTTGTGGAAAGGACGA |
| P5 – CGA stagger | AATGATACGGCGACCACCGAGATCTACACTCTTTCCCTACACGACGCTCTTCCGATCTCGATCTTGTGGAAAGGACGA |
| P5 – ACGA stagger | AATGATACGGCGACCACCGAGATCTACACTCTTTCCCTACACGACGCTCTTCCGATCTACGATCTTGTGGAAAGGACGA |
| P5 – CTAGAA stagger | AATGATACGGCGACCACCGAGATCTACACTCTTTCCCTACACGACGCTCTTCCGATCTCTAGAATCTTGTGGAAAGGACGA |
| P5 – GACGACA stagger | AATGATACGGCGACCACCGAGATCTACACTCTTTCCCTACACGACGCTCTTCCGATCTGACGACATCTTGTGGAAAGGACGA |
| P5 – TGGACACA stagger | AATGATACGGCGACCACCGAGATCTACACTCTTTCCCTACACGACGCTCTTCCGATCTTGGACACATCTTGTGGAAAGGACGA |
| P7 – Index AATCCAGC Primed 0 A | CAAGCAGAAGACGGCATACGAGATGCTGGATTGTGACTGGAGTTCAGACGTGTGCTCTTCCGATCTTCTACTATTCTTTCCCCTGCACTGT |
| P7 – Index CCGAGTTA  Primed 0 B | CAAGCAGAAGACGGCATACGAGATTAACTCGGGTGACTGGAGTTCAGACGTGTGCTCTTCCGATCTTCTACTATTCTTTCCCCTGCACTGT |
| P7 – Index AACTGTTA  Primed 0 C | CAAGCAGAAGACGGCATACGAGATTAACAGTTGTGACTGGAGTTCAGACGTGTGCTCTTCCGATCTTCTACTATTCTTTCCCCTGCACTGT |
| P7 – Index TTGAGTAT  Primed 7 A | CAAGCAGAAGACGGCATACGAGATATACTCAAGTGACTGGAGTTCAGACGTGTGCTCTTCCGATCTTCTACTATTCTTTCCCCTGCACTGT |
| P7 – Index TTCACAGC  Primed 7 B | CAAGCAGAAGACGGCATACGAGATGCTGAGAAGTGACTGGAGTTCAGACGTGTGCTCTTCCGATCTTCTACTATTCTTTCCCCTGCACTGT |
| P7 – Index CCTCCAAT  Primed 7 C | CAAGCAGAAGACGGCATACGAGATATTGGAGGGTGACTGGAGTTCAGACGTGTGCTCTTCCGATCTTCTACTATTCTTTCCCCTGCACTGT |
| P7 – Index GGTCACCG  2i GFP+ A | CAAGCAGAAGACGGCATACGAGATCGGTGACCGTGACTGGAGTTCAGACGTGTGCTCTTCCGATCTTCTACTATTCTTTCCCCTGCACTGT |
| P7 – Index TTAGACTA  2i GFP+ B | CAAGCAGAAGACGGCATACGAGATTAGTCTAAGTGACTGGAGTTCAGACGTGTGCTCTTCCGATCTTCTACTATTCTTTCCCCTGCACTGT |
| P7 – Index CCTCTGTA  2i GFP+ C | CAAGCAGAAGACGGCATACGAGATTACAGAGGGTGACTGGAGTTCAGACGTGTGCTCTTCCGATCTTCTACTATTCTTTCCCCTGCACTGT |
| P7 – Index TTGACAAT  2i GFP- A | CAAGCAGAAGACGGCATACGAGATATTGTCAAGTGACTGGAGTTCAGACGTGTGCTCTTCCGATCTTCTACTATTCTTTCCCCTGCACTG |
| P7 – Index AAGACATA  2i GFP- B | CAAGCAGAAGACGGCATACGAGATTATGTCTTGTGACTGGAGTTCAGACGTGTGCTCTTCCGATCTTCTACTATTCTTTCCCCTGCACTGT |
| P7 – Index AATCCAAT  2i GFP- C | CAAGCAGAAGACGGCATACGAGATATTGGATTGTGACTGGAGTTCAGACGTGTGCTCTTCCGATCTTCTACTATTCTTTCCCCTGCACTGT |

**Supplementary Table 2. List of primers used for generating amplicon libraries.** For P5 primers a mixture of primers was used, with each P5 primer differing in the length of the stagger region. The stagger region is placed immediately upstream of the plasmid specific sequence of the primer and is required to generate sequence diversity required when undertaking Illumina based next-generation sequencing – the sequence of the stagger is indicated and highlighted in red in the P5 primer sequence. For the P7 primers the 8bp sequence of the index is shown a long with the sample each index represents – highlighted in red is the index region in the P7 primer sequence.

**Supplementary Table 3. List of primers used for targeted bisulfite sequencing**. Regions underlined correspond to the sequencing adapter. The region of the indexed library amplification reverse primer highlighted in bold corresponds to the index.

|  | **Forward Primer** |
| --- | --- |
| **D1_17** | CTACACGACGCTCTTCCGATCTGGTTAAGTTAGGTTTTTTATTTAGTT |
| **D3_4** | CTACACGACGCTCTTCCGATCTTTGAGGGAATGAGATTAGTGAAT |
| **D4_6** | CTACACGACGCTCTTCCGATCTGGAAGGTAGATTAAAGAAAGTGTA |
| **D5_6** | CTACACGACGCTCTTCCGATCTGAGTTAATGGGTTTTGTGTTAAGT |
| **D6_14** | CTACACGACGCTCTTCCGATCTATGTAGGGGATAATTGATTGTAGAG |
| **D8_16** | CTACACGACGCTCTTCCGATCTATAGTTGATGGAGGTAGGAGAGT |
| **D9_10** | CTACACGACGCTCTTCCGATCTGTTAATTTTTATGTAGGGATGTAG |
| **Igf2r_18** | CTACACGACGCTCTTCCGATCTGTTTTATAGGAGGGAAGGGTTT |
| **IAP1_10** | CTACACGACGCTCTTCCGATCTTTGTTGAAATGGAAGGGTTAG |
| **Indexing library amplification** | AATGATACGGCGACCACCGAGATCTACACTCTTTCCCTACACGACGCTCTTCCGATCT |

|  | **Reverse Primer** |
| --- | --- |
| **D1_17** | CAGACGTGTGCTCTTCCGATCTACCTCTATAATAATAAAAACCCTC |
| **D3_4** | CAGACGTGTGCTCTTCCGATCTCTCTTCTCAAATCCCTACACATAA |
| **D4_6** | CAGACGTGTGCTCTTCCGATCTCCCTAAACTCTACCTTTAACCTT |
| **D5_6** | CAGACGTGTGCTCTTCCGATCTATACCCTCAACAACTACCACTTAA |
| **D6_14** | CAGACGTGTGCTCTTCCGATCTAATCTATTCCATCACTCCCCTT |
| **D8_16** | CAGACGTGTGCTCTTCCGATCTTCACAAATTAAATAAAAACCTATACC |
| **D9_10** | CAGACGTGTGCTCTTCCGATCTCAACTATCCTACTATAATCCAAA |
| **Igf2r_18** | CAGACGTGTGCTCTTCCGATCTCCTCCCTTCTCCTCTTACTAAC |
| **IAP1_10** | CAGACGTGTGCTCTTCCGATCTACTACAACCAATCAAAAAATAACAC |
| **Indexing library amplification** | CAAGCAGAAGACGGCATACGAGAT**TACAAGTT**GTGACTGGAGTTCAGACGTGTGCTCTTCCGATCT |
