# Supplementary material for: An shRNA kinase screen identifies regulators of UHRF1 stability and activity in mouse embryonic stem cells
Source: Epigenetics. 2022 Mar 24;17(12):1590–607. doi: 10.1080/15592294.2022.2044126 (PMC9621053; doi:10.1080/15592294.2022.2044126)

Supplementary Figure 1.

A

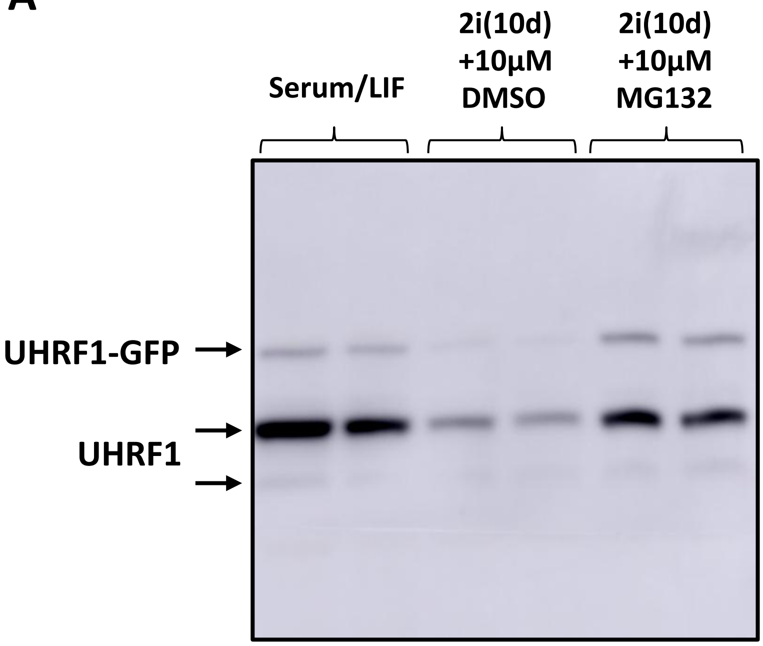

B

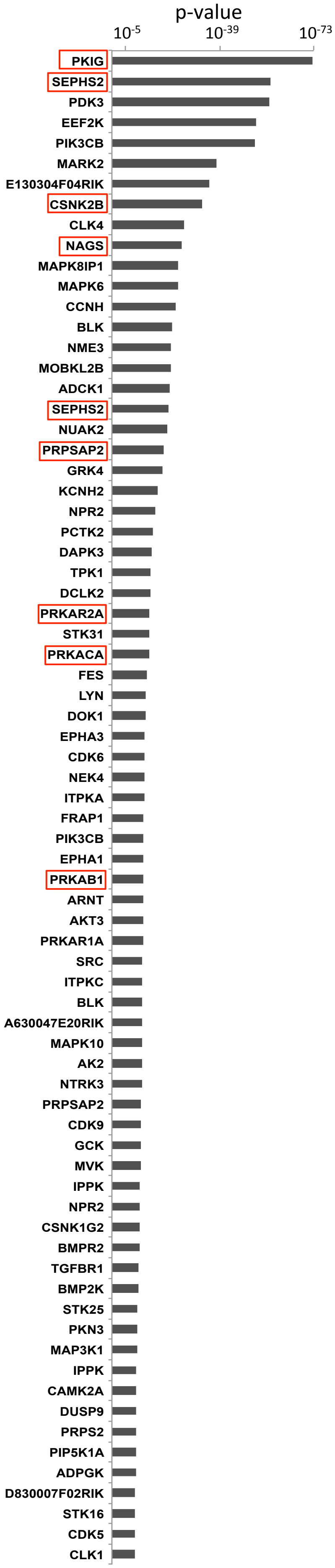

C

Candidate genes with >=2 shRNA identified in the screen

4930444A02RIK	FES	PIK3CB
AFF1	FRAP1	PIM1
AK3	GSK3B	PIP5K1A
AKT3	IPPK	PIP5K1B
ARAF	ITPKA	PIP5K3
AXL	KCNH2	PODXL
BLK	LIMK1	PRPSAP2
BMPR2	MAP3K14	PSKH1
CABC1	MAP3K7IP1	RFK
CCNH	MAPK8	RIPK2
CKS1B	MELK	SEPHS1
CSNK1G2	NEK4	SEPHS2
CSNK2A1	NME3	SGMS1
CSNK2B	NME4	SH3KBP1
EPHA1	NPAS3	STK11
EPHA6	NPR2	STK19
EPHB2	NUCKS1	STK31
EPHB4	PCTK1	TLK1
		TRIB2

Supplementary Figure 2.

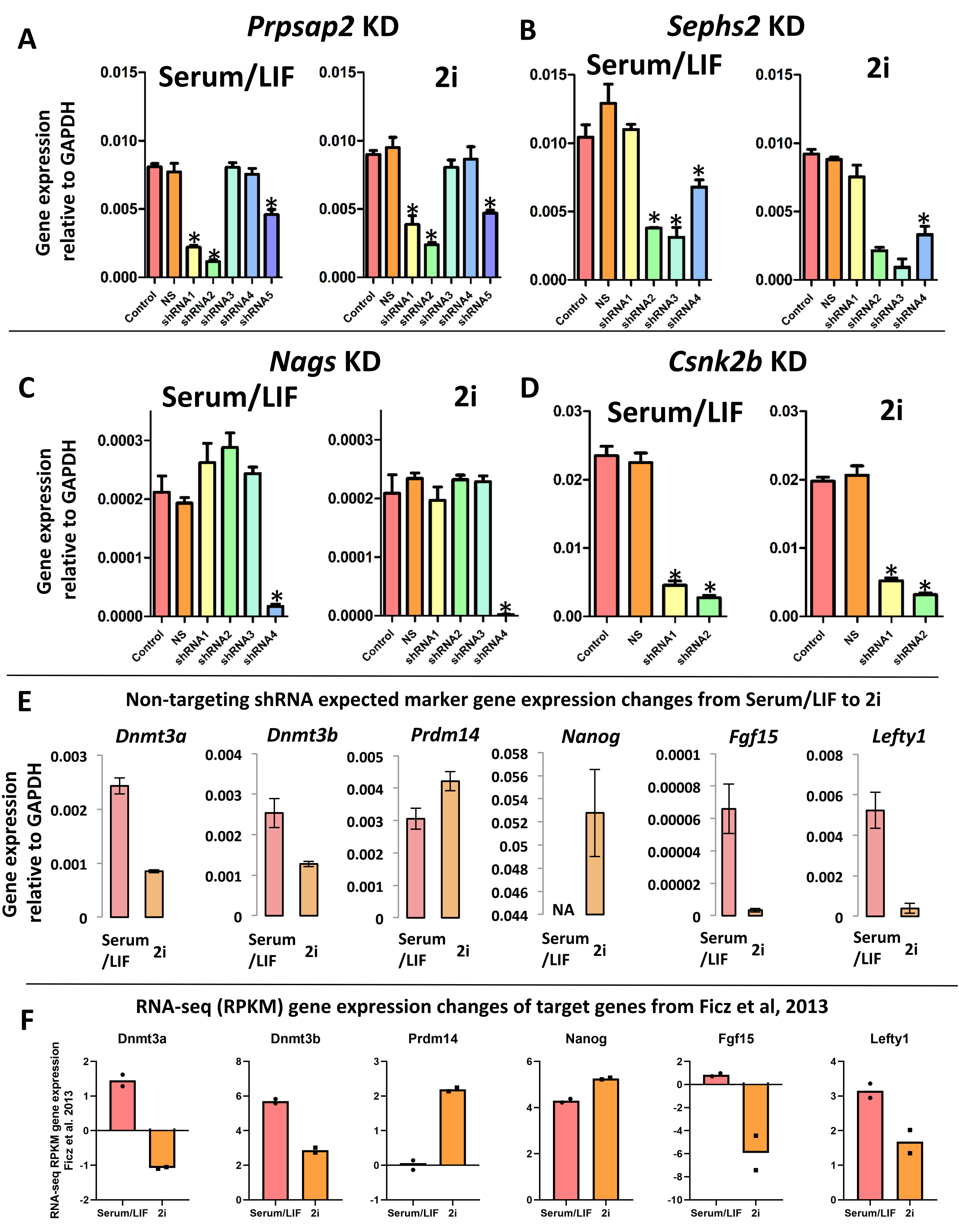

Supplementary Figure 3.

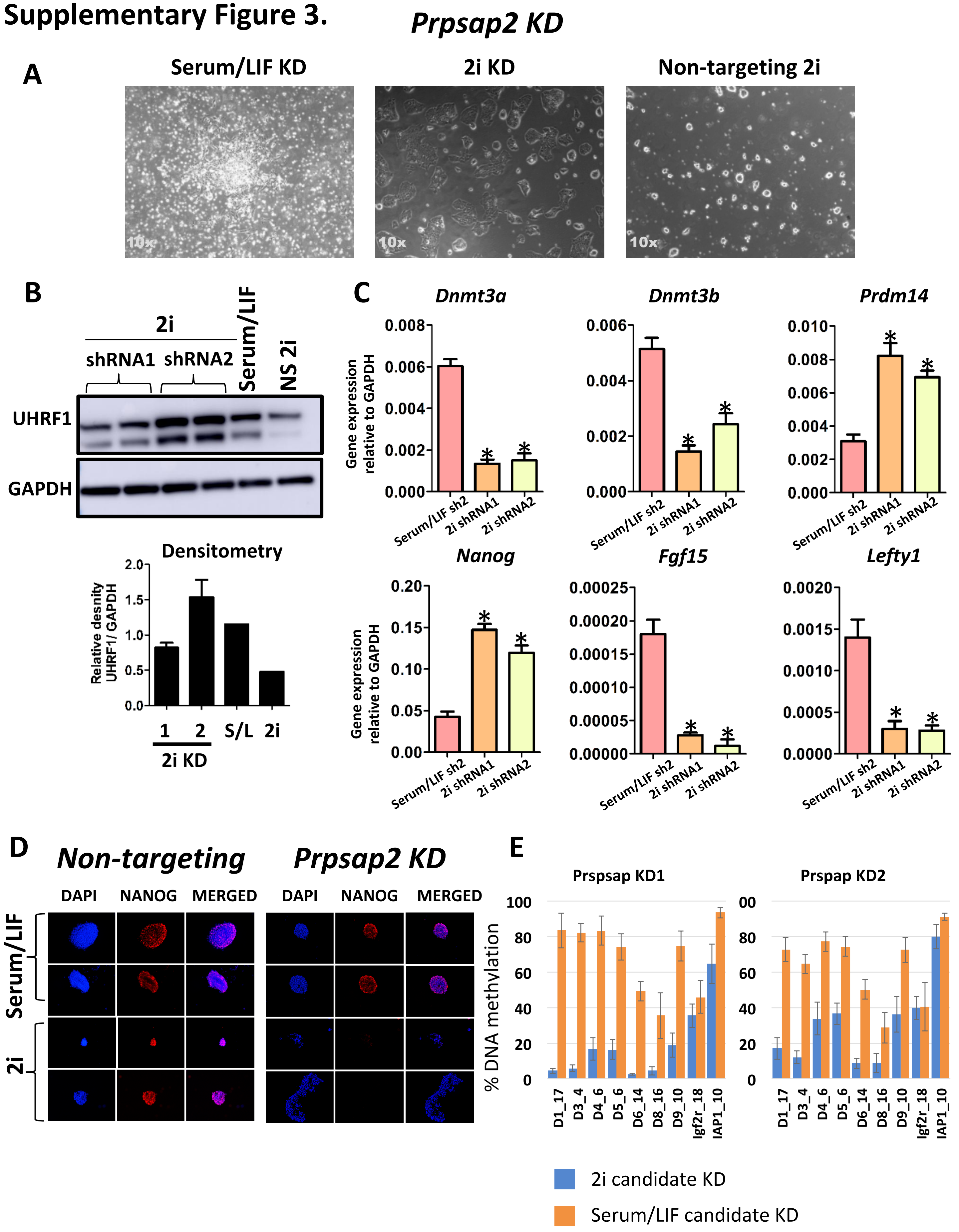

Supplementary Figure 4.

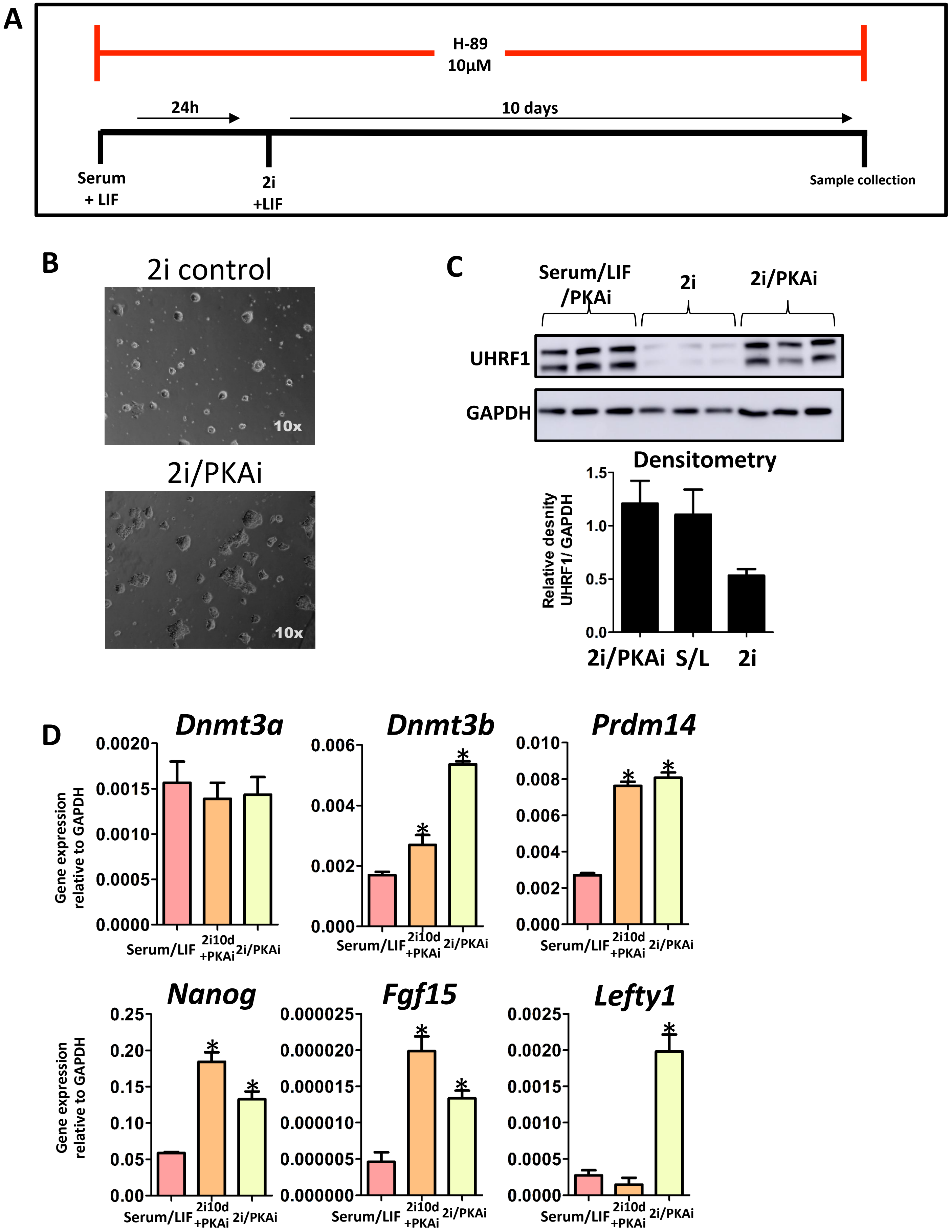

Supplementary Figure 5.

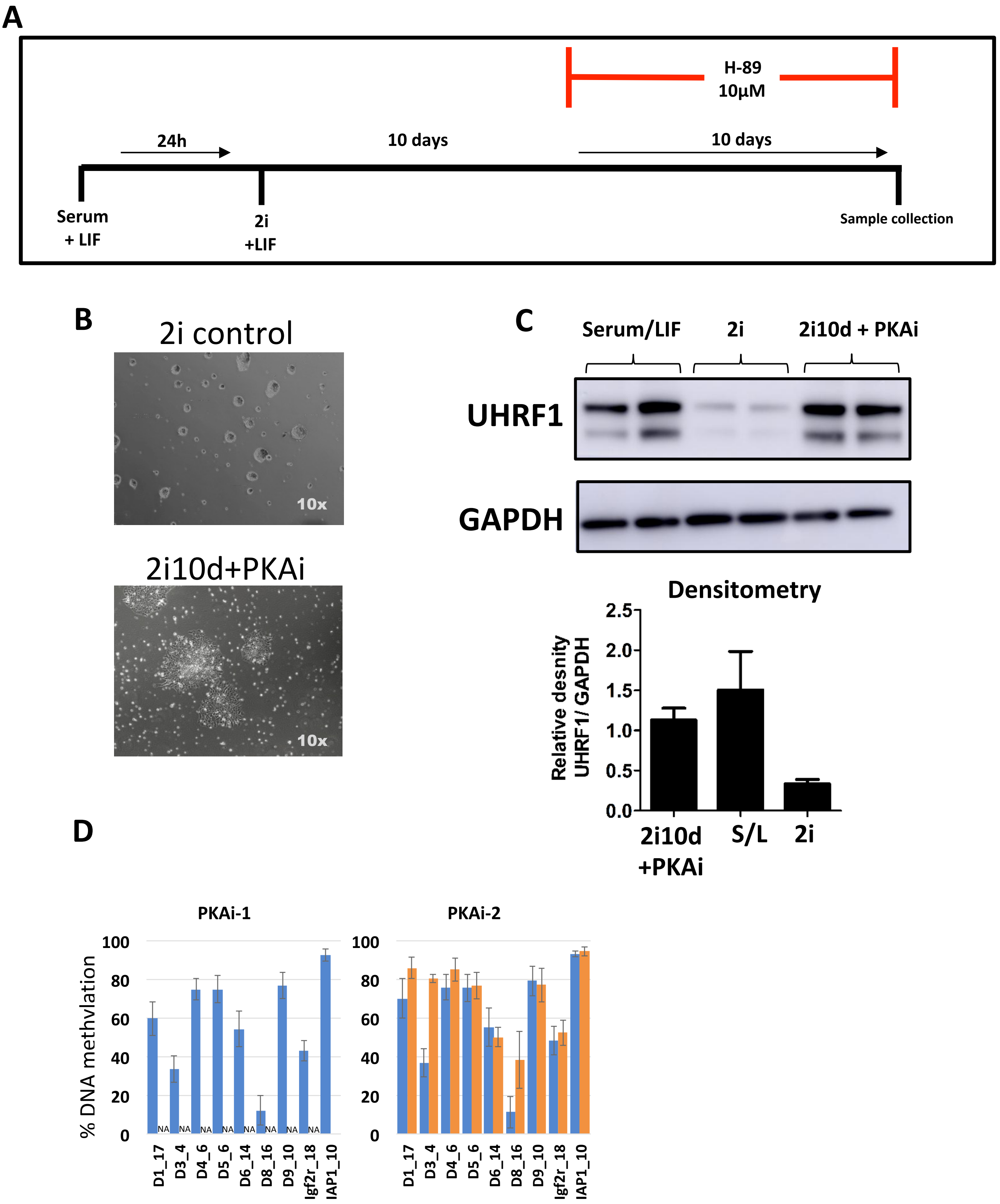

Supplementary Figure 6.

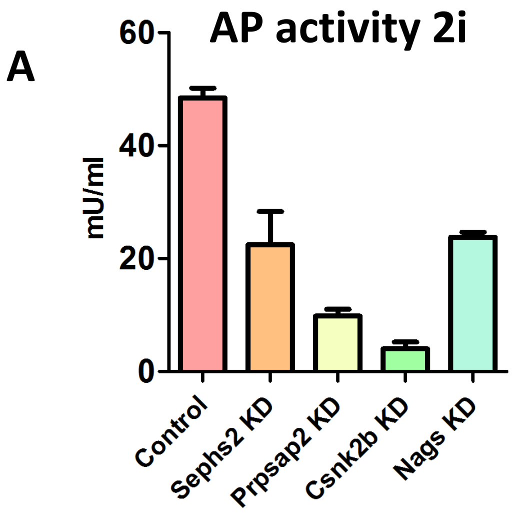

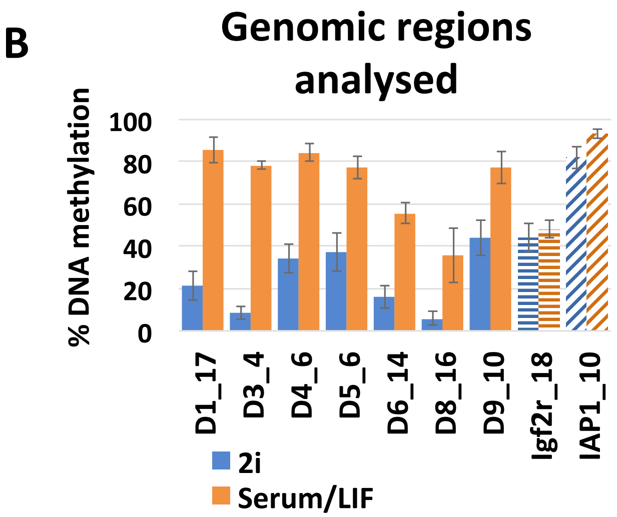

Supplement: Supplemental Material [file KEPI_A_2044126_SM0147.zip › Rushton_supplementary/SupplementaryFiguresRushton.pdf]
