# Supplementary material for: An shRNA kinase screen identifies regulators of UHRF1 stability and activity in mouse embryonic stem cells
Source: Epigenetics. 2022 Mar 24;17(12):1590–607. doi: 10.1080/15592294.2022.2044126 (PMC9621053; doi:10.1080/15592294.2022.2044126)
Supplement: Supplemental Material [file KEPI_A_2044126_SM0147.zip › Rushton_supplementary/Targeted bisulfite seq analysis.docx]

**Targeted bisulfite analysis**

**Step 1** Fastq files were transferred to Apocrita (QMUL HPC)

Example of data from one amplicon:

#read1="Serum-control_R1.fq.gz"

#read2="Serum-control_R2.fq.gz"

**Step 2** Prepare custom genome

Need all the amplicons listed as fasta file, an example below (use rtf for this to avoid interruptions in text; I called this **rushton.fa**):

>CpG-Igf2r-chr17

AATTATTACGTAGATATTTTGGGGAATTGAGGTAAGTTAGGGTTTTTCGCGTGAAGCGCGGTAATGCGAGGGGAGGATTTTGTAGATGAGGGTAGGATTTCGTTGTAAGGGGAGGATTTTACGCGTTAGAGGATTTCGTAAA

GGAAGGGTTTTATAGGAGGGAAGGGTTTTACGCGAGGTGAGGGTTTTATTGATTCGGTAGTTCGAGGGTTTCGATTAAGAGTTTTAGGTCGTTTAAAGGTTTTTTTACGCGAGATTTAGCGTTAGGGTGAAGATTTTTGGGT

TATAAGAAAATTTAGTATTAGGGTGTTTTATTGTTTATTAGTGTTTTGAATTATACGAGGGTCGATAGGGTCGGTAGAAAGTCGAAAGTCGCGTTGTCGTTATGTCGCGTTAGTAAGAGGAGAAGGGAGGGGGGCGTTTAGC

GCGGGTCGATTGTTGTATTGGGGGGGGGGGGGGTTTACGGGCGATTTAGAGTACGAGGGTGTTACGTTGCGTAAGGGGAGTAGGGGTTCGCGTTAGGGTATTACGTTGTTGGAGAGTTGGGGGGGGGGGTGGAGATCGGAGG

ATTTTTATACGATTTGATTCGCGGTTTGCGGGGTAGGGGGAAGGTAA

**Step 3** Need a list of the files names for the script below (this is **fastq_list.txt**)

example of my files:

2i-control_R1.fq.gz

2i-CSNK2B-KD-1_R1.fq.gz

2i-CSNK2B-KD-2_R1.fq.gz

2i-NAGS-KD-1_R1.fq.gz

2i-NAGS-KD-2_R1.fq.gz

2i-PKAi-1_R1.fq.gz

2i-PKAi-2_R1.fq.gz

2i-PRPSAP2-KD-1_R1.fq.gz

2i-PRPSAP2-KD-2_R1.fq.gz

2i-SEPHS2-KD-1_R1.fq.gz

2i-SEPHS2-KD-2_R1.fq.gz

Serum-control_R1.fq.gz

Serum-CSNK2B-KD-1_R1.fq.gz

Serum-CSNK2B-KD-2_R1.fq.gz

Serum-NAGS-KD-1_R1.fq.gz

Serum-NAGS-KD-2_R1.fq.gz

Serum-PKAi-1_R1.fq.gz

Serum-PKAi-2_R1.fq.gz

Serum-PRPSAP2-KD-1_R1.fq.gz

Serum-PRPSAP2-KD-2_R1.fq.gz

Serum-SEPHS2-KD-1_R1.fq.gz

Serum-SEPHS2-KD-2_R1.fq.gz

**Step 4** Run **targetedBSseqarraycustomgenome.sh**

#!/bin/sh

#$ -cwd

#$ -l h_rt=12:0:0

#$ -pe smp 2

#$ -l h_vmem=1G

#$ -N targeted_methylation_array

#$ -t 1-22

#####################

# Load modules

#####################

module load bowtie2/2.3.4

module load samtools/1.9

module load trimgalore

#####################

# Specify paths and filenames

#These are examples of names in the fastq_list:

#read1="Serum-control_R1.fq.gz"

#read2="Serum-control_R2.fq.gz"

#####################

fastqdir=/data/BCI-Ficz/Gabi/Rushtonetal/fastqrawfiles

outputdirectory=/data/BCI-Ficz/Gabi/Rushtonetal/output_dir

genome=/data/BCI-Ficz/Gabi/Rushtonetal/

#read list of files from fastq_list.txt

#run this job from the directory where fastq_list.txt is

file=$(sed -n "${SGE_TASK_ID}p" fastq_list.txt)

base_name=$(echo $file | sed s/_R1.fq.gz//)

# Trim

trim_galore --dont_gzip --fastqc_args "-o QC" --paired $fastqdir/${base_name}_R1.fq.gz $fastqdir/${base_name}_R2.fq.gz

#Bismark genome preparation

/data/BCI-Ficz/Gabi/Rushtonetal/bismark_v0.22.1/bismark_genome_preparation ${genome}

# Bismark alignment to produce bam files

/data/BCI-Ficz/Gabi/Rushtonetal/bismark_v0.22.1/bismark --score_min L,0,-6 --output_dir ${outputdirectory} --genome ${genome} \

-1 ${base_name}_R1_val_1.fq \

-2 ${base_name}_R2_val_2.fq

# Bismark methylation extractor

/data/BCI-Ficz/Gabi/Rushtonetal/bismark_v0.22.1/bismark_methylation_extractor -p --bedGraph --buffer_size 10G --cytosine_report \

--genome_folder ${genome} ${outputdirectory}/${base_name}_R1_val_1_bismark_bt2_pe.bam

samtools sort -o ${base_name}_sorted.bam ${outputdirectory}/${base_name}_R1_val_1_bismark_bt2_pe.bam

samtools index ${base_name}_sorted.bam

**Step 5** Extract methylation data

An example of the files that are resulting from Step 4:

128K -rw-rw---- 1 hfw821 BCI-Ficz 3.0K Nov 1 22:41 Serum-control_R1.fq.gz_trimming_report.txt

128K -rw-rw---- 1 hfw821 BCI-Ficz 1.6K Nov 1 22:42 Serum-control_R1_val_1_bismark_bt2_pe.bedGraph.gz

128K -rw-rw---- 1 hfw821 BCI-Ficz 1.9K Nov 1 22:42 Serum-control_R1_val_1_bismark_bt2_pe.bismark.cov.gz

128K -rw-rw---- 1 hfw821 BCI-Ficz 14K Nov 1 22:42 Serum-control_R1_val_1_bismark_bt2_pe.CpG_report.txt

128K -rw-rw---- 1 hfw821 BCI-Ficz 13K Nov 1 22:42 Serum-control_R1_val_1_bismark_bt2_pe.M-bias.txt

512 -rw-rw---- 1 hfw821 BCI-Ficz 826 Nov 1 22:42 Serum-control_R1_val_1_bismark_bt2_pe_splitting_report.txt

9.7M -rw-rw---- 1 hfw821 BCI-Ficz 9.7M Nov 1 22:41 Serum-control_R1_val_1.fq

128K -rw-rw---- 1 hfw821 BCI-Ficz 3.2K Nov 1 22:41 Serum-control_R2.fq.gz_trimming_report.txt

9.7M -rw-rw---- 1 hfw821 BCI-Ficz 9.6M Nov 1 22:41 Serum-control_R2_val_2.fq

3.8M -rw-rw---- 1 hfw821 BCI-Ficz 3.8M Nov 1 22:42 Serum-control_sorted.bam

512 -rw-rw---- 1 hfw821 BCI-Ficz 744 Nov 1 22:42 Serum-control_sorted.bam.bai

Script to extract methylation data in R using the cov files:

2i-control_R1_val_1_bismark_bt2_pe.bismark.cov

2i-CSNK2B-KD-1_R1_val_1_bismark_bt2_pe.bismark.cov

2i-CSNK2B-KD-2_R1_val_1_bismark_bt2_pe.bismark.cov

2i-NAGS-KD-1_R1_val_1_bismark_bt2_pe.bismark.cov

2i-NAGS-KD-2_R1_val_1_bismark_bt2_pe.bismark.cov

2i-PKAi-1_R1_val_1_bismark_bt2_pe.bismark.cov

2i-PKAi-2_R1_val_1_bismark_bt2_pe.bismark.cov

2i-PRPSAP2-KD-1_R1_val_1_bismark_bt2_pe.bismark.cov

2i-PRPSAP2-KD-2_R1_val_1_bismark_bt2_pe.bismark.cov

2i-SEPHS2-KD-1_R1_val_1_bismark_bt2_pe.bismark.cov

2i-SEPHS2-KD-2_R1_val_1_bismark_bt2_pe.bismark.cov

Serum-control_R1_val_1_bismark_bt2_pe.bismark.cov

Serum-CSNK2B-KD-1_R1_val_1_bismark_bt2_pe.bismark.cov

Serum-CSNK2B-KD-2_R1_val_1_bismark_bt2_pe.bismark.cov

Serum-NAGS-KD-1_R1_val_1_bismark_bt2_pe.bismark.cov

Serum-NAGS-KD-2_R1_val_1_bismark_bt2_pe.bismark.cov

Serum-PKAi-2_R1_val_1_bismark_bt2_pe.bismark.cov

Serum-PRPSAP2-KD-1_R1_val_1_bismark_bt2_pe.bismark.cov

Serum-PRPSAP2-KD-2_R1_val_1_bismark_bt2_pe.bismark.cov

Serum-SEPHS2-KD-1_R1_val_1_bismark_bt2_pe.bismark.cov

Serum-SEPHS2-KD-2_R1_val_1_bismark_bt2_pe.bismark.cov

setwd('~/Documents/Data_analysis/Targeted-BSsequencing/FolderName')

##get files

files = list.files()

cov.files = files[grep('.cov',files)]

##get data

data = list()

for (i in 1:length(cov.files)) {

data[[i]] = read.delim(cov.files[i],header=F)

colnames(data[[i]]) = c('amp','start','end','met','n.met','n.unmet')

}

names(data) = gsub("_R1_val.*","",cov.files)

##make coverage table

get.cov = function(x) {

total = x$n.met+x$n.unmet

cov = tapply(total,x$amp, median)

return(cov)

}

coverage = lapply(data, get.cov)

for (i in 1:length(coverage)) {

temp.df = data.frame(names(coverage[[i]]),coverage[[i]])

colnames(temp.df) = c('amp',names(coverage)[i])

if (i==1) {

cov.df = temp.df

} else {

cov.df = merge(cov.df,temp.df,by='amp',all=TRUE)

}

}

##filter data

f.data = lapply(data, function(x) x[x$n.met+x$n.unmet>=100,])

mismapped = function(x) {

total = x$n.met+x$n.unmet

remove = numeric()

for (a in unique(x$amp)) {

med = median(total[x$amp==a])

remove = c(remove, which(x$amp==a & total<0.2*med))

}

if (length(remove)>0) {

return(x[-remove,])

} else {

return(x)

}

}

f.data = lapply(f.data, mismapped)

##compile methylation data

for (i in 1:length(f.data)) {

temp.df = data.frame(paste(f.data[[i]]$amp,f.data[[i]]$start,sep='_'), f.data[[i]]$met)

colnames(temp.df) = c('amp_pos',names(f.data)[i])

if (i==1) {

met.df = temp.df

} else {

met.df = merge(met.df, temp.df, by='amp_pos',all=TRUE)

}

}

write.table(met.df, file="RushtonTargetedAnalysis.txt", quote=F, sep='\t', row.names=T)

Code written by Dr. Miguel Branco
